# Large-scale randomized double-blind field clinical trial for safety and efficacy assessment of the DNA vaccine Neoleish against canine leishmaniasis

**DOI:** 10.1371/journal.pntd.0012707

**Published:** 2025-11-03

**Authors:** Llum Páez, Alberto Parra, Elena Sotelo, Iria Taboada, Raquel López, Paula Segade, María del Mar Boente, María José Rivas, Lidia Da Rocha, Ana Alonso, Pedro José Alcolea, Eugenia Puentes

**Affiliations:** 1 CZ Vaccines, Zendal Group, O Porriño, Pontevedra, Spain; 2 Centro de Investigaciones Biológicas Margarita Salas, Consejo Superior de Investigaciones Científicas (CIB-CSIC), Madrid, Spain; Bose Institute, INDIA

## Abstract

Canine leishmaniasis is a vectorial zoonotic disease caused by the obligate intracellular trypanosomatid parasite *Leishmania infantum*. This chronic disease is characterized by a variable combination of cutaneous and visceral clinical signs. Despite the availability of insecticides and first-line drug therapies, prevalence remains high in many areas fundamentally distributed in the Mediterranean basin and Brazil. The development of a vaccine against leishmaniasis is a challenging objective in veterinary medicine due to the parasite’s life cycle complexity, resistance, relapses, and toxicity of the currently available drugs. Vaccination against canine leishmaniasis intends to decrease the parasite burden and the risk of clinical disease. Neoleish is a third generation DNA vaccine based on the *L. infantum* LACK gene encoding the 36 kDa protein, analogue of the receptor of the activated protein kinase C (LACK/p36) included in the antibiotic resistance-free plasmid pPAL. Once safety and efficacy of this intranasally delivered vaccine was confirmed in the preclinical phase, this randomized double-blind field trial was performed to assess safety and efficacy of the Neoleish vaccine. It was assessed by exposing 361 healthy naïve dogs to natural *L. infantum* infection during two consecutive transmission seasons in three endemic areas of Spain. 361 dogs were randomly split into two treatment groups (181 vaccinated and 180 placebo). The primary safety endpoint was the absence of serious local and/or systemic adverse events and/or deaths attributable to vaccination. Neoleish demonstrated a high safety profile. No signs of shock, local or systemic reactions were observed even after the administration of an overdose (10x) of Neoleish followed by a repeated dose. The absence of Neoleish interference with the ELISA and IFAT serological tests was confirmed after repeated vaccination. Regarding efficacy, Neoleish vaccine affected the progression of parasite multiplication in bone marrow and peripheral blood, showing a statistically significant reduction of parasite load in vaccinated animals at the end of the study. In summary, the contributions of Neoleish, a vaccine with a very high safety profile, to canine leishmaniasis prevention are: i) The odds-ratio for detectable parasites in blood is 3.5 for vaccinated dogs compared to non-vaccinated dogs; if detected, ~86% lower parasitemia levels are expected; ii) A vaccinated dog has a 2-fold lower risk of developing active infection compared to a non-vaccinated dog; in case positive, the parasite load in bone marrow is expected to be ~96% lower; iii) A 3-fold lower risk (~72.7%) of developing clinical disease is expected in a vaccinated compared to a non-vaccinated dog.

## Authors’ summary

Vaccines are valuable prevention weapons against infectious diseases, specially those showing complex and efficient transmission patterns and limited efficacy of drug treatment and other control measures. Dogs are the main reservoirs of *Leishmania infantum*, a protozoan parasite responsible for leishmaniasis in different mammalian species including humans. Canine leishmaniasis is a public health problem. Vaccines are required because prevalence in endemic areas remains high despite available drugs and insecticides. *L. infantum* is an obligate intracellular parasite, so the cellular immune response is essential to control the disease. DNA vaccines consist of the administration of antigen-coding genes in an appropriate DNA vector such a plasmid. The vaccine is internalized and the LACK gene expressed, in first place, in cells of the vaccinated animal at the inoculation site eliciting specific activation of the adaptative immune response. Neoleish is a DNA vaccine against canine leishmaniasis based on the gene encoding the activated protein kinase C receptor analogue from *L. infantum* (LACK) included in the pPAL mammalian expression plasmid vector. After successful safety and efficacy preclinical assessment, a double-blind randomized field trial confirmed that this DNA vaccine is able to reduce the risk of infection (3.5-fold in blood and 2-fold in bone marrow) and clinical disease (~72.7%), as well as to diminish the parasite burden (~86% in blood and ~96% in bone marrow). The vaccine is fully safe, including in infected dogs, and does not interact with ELISA and IFAT serological test diagnosis.

## Introduction

Canine leishmaniasis (CanL) is a disease caused by intracellular protozoan parasites of the species *Leishmania infantum* (Kinetoplastida:Trypanosomatidae). The parasite’s life cycle is digenetic alternating the promastigote stage in the gut of the phlebotomine sand fly vector (Diptera:Psychodidae) and the amastigote stage in phagocytes of the mammalian host. There is a wide spectrum of clinical manifestations ranging from subpatent asymptomatic infections to chronic cutaneous ulcers and fatal visceral infection. CanL is broadly distributed in most tropical and Mediterranean countries [1]. In this region dogs are the main reservoir of the parasite, which is transmitted to other dogs and humans by the bite of phlebotomine sand flies of the *Phlebotomus (Larroussius)* subgenus. Despite the availability of several topical insecticide preparations with good trial data for dogs (reviewed by [2]), these products cannot prevent all potential infectious sand fly bites, and there is still a need for further control measures. Current treatments require long-term therapy and present adverse side effects or relapse due to parasite resistance. Consequently, the development of a vaccine against leishmaniasis is a long-term goal in veterinary medicine. There are at least two main objectives of vaccination against CanL: the risk reduction of suffering from clinical disease, and risk reduction of subsequent horizontal parasite transmission, by reducing incidence and decreasing parasite burden in infected dogs.

There are several vaccines against *L. infantum* and other *Leishmania* species under preclinical development in murine models. Although first generation vaccines, or whole-organism-based vaccines, are still being tested, including killed and inactivated parasite vaccines (e.g., [3]), most are second-generation vaccines, or subunit vaccines, that frequently consist of chimeras containing multi-epitopes and require liposomes [4], adjuvants [5], nanoparticles [6], or virus-like particles (VLPs) [7] for administration. Third generation vaccines, or nucleic acid vaccines, comprise RNA and DNA vaccines. DNA vaccines can be administered as naked recombinant plasmid DNA or as DNA vaccine constructs vehicled into viral vectors. DNA vaccines against *Leishmania* spp. are under testing. Most are based on plasmids with antibiotic resistance genes [8–10] and some without them [11], although vaccines based in viral vectors such as ChAd63-KH have also been tested. ChAd63-HK is based on an adenoviral vector and the HASPB and KMP-11 antigens [12]. DNA internalization in cells is an issue of DNA vaccines that can be solved by several methods such as electroporation or needle-free system. Also, employment of the intranasal route favors uptake by the abundant antigen-presenting cells present in this mucosa, which is rich in toll-like receptor 9 (TLR9) [13]. TLR9 has affinity for unmethylated CpG oligodeoxynucleotide motifs such as those the pPAL vector contains [14].

Four vaccines against canine leishmaniasis were authorized before Neoleish. The first was Leishmune, which was authorized in Brazil in 2004 and withdrawn 10 years later. Leish-Tec is a second generation vaccine that consists of the A2 protein and was withdrawn in 2023. The second generation vaccine Canileish (*L. infantum* Excreted-Secreted Antigen (LiESP) plus QA21 adjuvant) was the first vaccine against canine leishmaniasis authorized in Europe and was withdrawn in 2023. Letifend is a second generation vaccine currently used in Europe. This vaccine is based on protein Q, a chimeric protein encoding five fragments from four *L. infantum* proteins (reviewed by [15]). The most recently approved vaccine by the European Commission (EC) based on the European Medicines Agency’s (EMA) opinion [16], is Neoleish, which consists of the activated protein kinase C receptor analogue (LACK) cloned into the pPAL vector. The LACK protein belongs to the large family of WD 40 repeat proteins. This family has been found only in eukaryotes and is involved in numerous regulatory functions. The LACK antigen contains peptide sequences that can bind sequences of vacuolar proteins involved in DNA replication and RNA synthesis. This suggests the involvement of these LACK determinants in the host response against the parasite through a hypothetical MHC-II-binding ability, possibly triggering cytokine production [17]. LACK induces high levels of protection against parasite infection in the BALB/c mouse model [18]. This protection is more than twice as high as that elicited by major parasite antigens such as soluble *Leishmania* antigen or the main surface protease gp63 [19]. LACK-tolerant transgenic mice obtained by transgene expression in the thymus exhibited both a diminished Th2 response and a healing phenotype acting through inhibitory signals for Th2 differentiation, unbalancing the delicate equilibrium between the two CD4^+^ cell subsets. Thus, T cells early activated against the single antigen play a pivotal role in directing the immune response to the entire parasite [17].

The LACK antigen administered as DNA vaccines protect BALB/c mice against *L. major* [20,21] and *L. infantum* [22,23], as well as Syrian hamsters against *L. infantum* [24] activating a specific Th1 response. LACK also protects Beagle dogs against experimental *L. infantum* challenge when administered as a DNA vaccine by the subcutaneous route [25–27]. Neoleish, is a third generation DNA vaccine based on the *L. infantum* LACK gene cloned in the mammalian expression plasmid vector pPAL. This vector is based on the CMV enhancer/promoter expression system and the *fab I* - triclosan selection system [28] and also acts as an adjuvant because it contains a CpG island [14]. 1 mL dose of vaccine contains 212.5-250 µg of supercoiled plasmid DNA in phosphate-buffered saline. No additional adjuvant or preservative is included in the vaccine composition. Neoleish is administered by the intranasal (IN) route in two 1 mL doses, 14 days apart.

A favourable safety profile was demonstrated when administered in a single dose and in repeated doses, as well as when a 10x overdose was administered followed by a repeated administration of a standard dose. In addition, repeated vaccine administration in infected dogs (PCR positive and/or seropositive) was followed up for at least one year, demonstrating that Neoleish was also safe in infected dogs and did not exacerbate the course of the disease. No post-vaccination shock or systemic or local reactions were observed in any of the trials performed. No significant increases in rectal temperature, negative effects on body weight, or hematological or biochemical deviations were observed. Biodistribution and persistence studies after an overdose and a standard repeated dose demonstrated no macroscopic or histological alterations, a short systemic distribution and a most frequent location in tissues close to the inoculation side (local lymph nodes and nasal mucosa). The vaccine was excreted in faeces, 24 hours after the first and second doses. Finally, the plasmid was not detected in any tissue at study termination, 91 days after the first dose [16].

The preclinical laboratory efficacy studies demonstrated that Neoleish vaccination triggered an immune response characterized by specific activation of T-cells in both peripheral blood and in relevant tissues for parasite replication, such as lymph nodes and spleen. This cell activation is accompanied by interferon-γ (IFN-γ) production [29], which is an indicator of Th1 response activation. Cellular activation has been proven to be long-lasting, and it has been demonstrated one year after vaccination, and revaccination 6 months after the primary vaccination course induces a detectable booster effect in specific T cell activation [16]. This immunological profile is related to the efficacy results demonstrated after experimental challenge [29]. A very high amount of promastigotes 10^8^ was administered to Beagle dogs by the intravenous route for experimental infection during Neoleish preclinical testing [29], which is much higher than in natural infection. The rationale for the higher amount of promastigotes employed in experimental challenge is to ensure infection success for preclinical vaccine efficacy testing because vector-free (i.e., artificial) infection is less efficient. For example, sand fly saliva is not present and infectivity is lower when cultured promastigotes are used in experimental infections (reviewed in [30]). With the optimized experimental infectious challenge of 10^8^ promastigotes, Neoleish has demonstrated significant reduction of parasite burden in bone marrow at different time points and in relevant tissues at the end of the study and significant reduction of the severity and clinical development of the disease. Following the clinical development of the Neoleish vaccine, the objective of this pivotal study was to evaluate the safety and efficacy of the plasmid DNA vaccine against CanL under real field conditions in a large representative population of dogs, exposed to natural infection during two transmission seasons. Selected dogs were located in open kennels situated in *Leishmania*-endemic areas of Spain. Hence, the objective of this veterinary clinical trial was to evaluate the safety and efficacy of the Neoleish vaccine in field conditions against infection with *L. infantum* in dogs.

## Methods

### Ethics statement

The study was compliant with the animal experimentation ethical standards (Directive 2010/63/EU), approved by the CZ Vaccines S.A.U. Ethics Committee, and subsequently authorized by the European Medicines Agency (389/ECV) and complied with the Good Clinical Practice (GCP) according to the recommendations of the Directive 81/852/EEC “Good Clinical Practice for the Conduct of Clinical Trials on Veterinary Medicinal Products in the European Union”, the VICH Guideline GL9 “Guideline on Good Clinical Practices” (CVMP/VICH/595/98-Final, July 2000), and the guidelines EMEA/CVMP/852/99-Final “Field trials with veterinary vaccines” and EMEA/CVMP/816/00-Final “Guideline on statistical principles for veterinary clinical trials”.

### Trial design

This study was designed as a double-blind trial, with parallel homogeneous groups (1:1, vaccinated:control), randomised and stratified in cohorts located in three different epidemiological areas (3 areas x 2 treatment groups). The trial was conducted in animal kennels located in epidemiologically active areas of CanL, with seroprevalence values higher than 8% [31], located in the provinces of Barcelona (B area), Cáceres (CC area) and Badajoz (BA area), where the presence of *Phlebotomus perniciosus* and *Phlebotomus ariasi*, the two most common *L. infantum* vectors in the Iberian Peninsula, were described [31,32]. The trial has been conducted in dogs older than 6 months of different breeds, weights, both sexes, not exposed (seronegative against full heterologous antigen assessed with direct commercial ELISAs-Ingezim/Civtest), not infected (negative qPCR form bone marrow) and non-symptomatic. In all cases, hunting dogs from different animal kennels located in rural or semi- urban areas that allow natural infection through the vector were selected. The duration of the study was two years, covering two full transmission cycles of the vector. Once the screening of suitable animals was completed, the distribution within each treatment group (A or B) was carried out randomly, on site, by each investigator at the moment of vaccination until there was a homogeneous number of animals per group in each kennel. Each animal in the vaccinated group was administered 2 doses of 1 mL, via the intranasal route (0.5 mL/nostril) of the Neoleish vaccine, with a 14-day interval. For intranasal use, the vaccine must be administered with a disposable syringe with nebulization cone so that it can be properly absorbed by the nasal mucosa without spilling a single drop. The control animals were inoculated with the same dose of PBS through the same route of administration. All animals were re-vaccinated 4 times with their respective treatments every 6 months after administering the recommended primo-vaccination scheme. The basic design of the trial is summarised in Table 1.

**Table 1:** Trial design.

Treatment group	No. dogs	No. dogs per area	First dose (T0)		Second dose (T14)		Re-vaccination (every 6 months: T194, T374, T554, T734)	
			Vaccine	Volume/ Route of administration	Vaccine	Volume/ Route of administration	Vaccine	Volume/ Route of administration
Vaccinated Group (GA)	181	Area 1 N = 61	NEOLEISH	1 mL/ IN*	NEOLEISH	1 mL/ IN	NEOLEISH	1 mL/ IN
		Area 2 N = 60						
		Area 3 N = 60						
Control/Placebo Group (GB)	180	Area 1 N = 60	Sterile PBS	1 mL/ IN	Sterile PBS	1 mL/ IN	Sterile PBS	1 mL/ IN
		Area 2 N = 60						
		Area 3 N = 60						

* IN: intranasal.

According to the Three Rs Principle (Replace, Reduce, and Refine), a minimum sample size was selected, consistent with the expected epidemiological conditions and the statistical power of the sampling. The selected sample size (total number of animals enrolled in the study) was 361 dogs, 181 vaccinated and 180 controls, distributed among 3 areas. The statistical justification of the selected sample size is described in the section Statistical Analysis below. The inclusion criteria were: animal kennels with seroprevalence ≥8% diagnosed with ELISA/PCR, approximately equal number of males and females, weight not specified (breed-dependent), > 6 months (minimum recommended age), no previous vaccine against *Leishmania* received, vaccinated and deparasitised according to the healthcare program established by the veterinary in charge of the animals, no previous exposure nor infection (seronegative to IFAT/ELISA and negative PCR in bone marrow), normal biochemical and blood profile, no clinical signs of leishmaniasis, and good body condition. All dogs enrolled had to be officially identified with a microchip.

**Fig 1:**
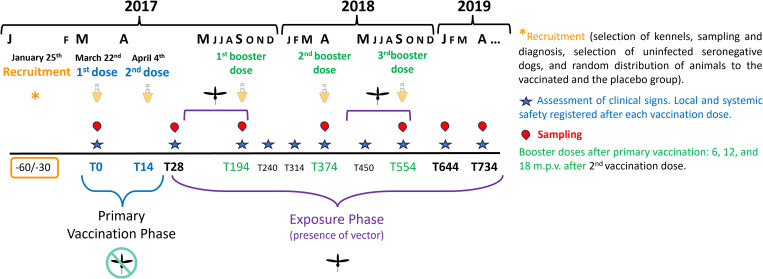
Schedule of the trial.

Sampling were carried out at T0 pv (before administration of the first dose), T28 pv (14 days after the second dose) and 194, 374, 554, 644, and 734 days after the first dose. The timeline of the study is represented in Fig 1. For efficacy assessment, samples of serum, whole blood, and bone marrow were taken.

As for the criteria to withdraw an animal, when an animal was diagnosed as infected (positive to qPCR) and clinically ill, the owners were advised to treat the affected animal or humanely slaughter the animal according to the current legal specifications.

### Clinical and paraclinical assessment of the disease

The assessment time points were: T0 (before administration of the first dose), T28 (14 days after the second dose), T194, T240, T314, T374, T450, T554, T644, and T734 d.p.v. (days post-vaccination) as detailed in Fig 1.

A daily clinical score (d.c.s.) was obtained for the sampling of each animal derived from the sum of each individual symptom using the scoring scale described in Table 2. Lastly, a global clinical score (g.c.s.) was obtained for each animal, obtained from the sum of each d.c.s.

**Table 2:** Clinical symptoms scoring system.

Clinical sign	Score
Low body condition	0 = absent, 1 = mild, 2 = moderate, 3 = severe
Anaemia	0 = absence, 1 = presence
Onychogryphosis	0 = absence, 1 = presence
Epistaxis	0 = absence, 1 = presence
Temporary muscle atrophy	0 = absence, 1 = presence
Conjunctivitis	0 = absent, 1 = mild, 2 = moderate, 3 = severe
Uveitis	0 = absent, 1 = mild, 2 = moderate, 3 = severe
Lymphadenopathy	0 = absent, 1 = 1–2 nodules, 2 = 2–4 nodules, 3 = 5–6 nodules, 4=>6 nodules
Alopecia	0 = absent, 1 = 1, 2=<5 mild, 3=>5 generalized high levels
Cutaneous ulcer	0 = absent, 1 = 1, 2=<5 mild, 3=>5 generalized high levels
Exfoliative dermatitis	0 = absent, 1 = 1 mild, 3 = multiple, 4 = generalized
Other signs	0 = absence, 1 = presence

Paraclinical assessment based on biochemistry profile was conducted. Each haematological/and biochemical change was assigned a score, with a simple scale: normal value = 0, mild increase/decrease = 1, moderate change = 2, serious change = 3. Mild change was defined as ≥ 5–10% from the expected value, moderate as ≥ 10–15% and serious as ≥ 15%. A paraclinical score (sum of the scores of all blood alterations) and cumulative score for the entire study (sum of d.c.s. and paraclinical score) were obtained for each animal at the time points specified above.

### Etiological diagnosis of canine leishmaniasis

#### Serological detection of *L. infantum* antibodies by indirect ELISA.

Two commercial antibody tests designed to detect serum IgG against *L. infantum* were sequentially used: INgezim *Leishmania* (Ingenasa, Spain) and Civtest Canis *Leishmania* (Hipra, Spain, later commercialized by Esteve, Spain as Leiscan). Both indirect ELISA tests use crude immunodominant *L. infantum* antigens for capturing specific IgGs. For detection, the INgezim test uses conjugation to a specific canine IgG monoclonal antibody, whereas Civtest uses a generic protein A/HRPO conjugate. INgezim was the test selected for seropositive assessment. All serum samples were tested in duplicate using this test, and those with doubtful results were re-tested in duplicate with the Civtest kit as a confirmatory test. For each ELISA test, the mean of absorbance readings was used to classify samples as positive, negative, or inconclusive by following the manufacturer’s instructions. Titer equivalence between Civtest and IFAT was obtained according to the manufacturer’s recommendations.

#### Parasitological detection and quantification by Real-Time PCR (qPCR) in bone marrow and peripheral blood.

Bone marrow and blood samples were repeatedly taken at different time points in order to detect and later quantify the presence of *L. infantum* DNA in biological samples. Bone marrow samples were extracted by puncturing the sternum and blood samples were obtained by saphenous venipuncture, both with disposable and single-use materials. Nucleic acid extractions were performed with the DNeasy Blood and Tissue Kit (Qiagen, Germany). Then real-time quantitative PCR (qPCR) was carried out as described [33,34].

For parasitological follow-up and quantification, all bone marrow samples were analyzed in duplicate as initial screening and those samples with mean Ct values <40 were re-tested, and the number of parasite/mL of sample was quantified for parasitological follow-up and parasite burden assessment. Finally, the dog samples showing positive amplification on bone marrow (Ct < 40), parasite burden in peripheral blood was additionally quantified by qPCR. For parasitological diagnosis (active infection), the threshold qPCR-positive result was set at Ct ≤ 27.00. Ct values comprised between 27 and 40 indicated subpatent infection.

qPCR was based on the method described by [33]. Briefly, the assay is based on a TaqMan real-time PCR type with MGB probe, which amplified a 120 bp region of a fragment of the *Leishmania* kinetoplast minicircle DNA. Each reaction also contains a pre-optimized commercial internal positive control (IPC) with pre-designed primers and TaqMan probe (*TaqMan Exogenous Internal Positive Control Reagents (IPC), Thermofisher*). For parasite quantification, spiked PCR negative biological samples (b.m. or blood) with known number of parasites/mL of sample, ranging from 1.8x10^4^ to 1.8x10^-1^ parasites/mL, in duplicate, were used as a linear calibrator by the comparative Ct method, allowing the quantification of the number of parasites per mL of sample. The calibrator was prepared from the *L. infantum* strain MCAN/ES/MON1/Z001.

#### Classification of the infection status of the dogs.

In accordance with previous recommendations [35–37] the classification of the dogs’ *Leishmania* status was determined using the results parasitological detection results (qPCR in bone marrow), serological detection (indirect ELISA tests) and the presence or absence of clinical/para-clinical signs typically described in leishmaniasis (Table 3) at each clinical assessment point upon study completion. It is important to note that infection is not equivalent to disease in the case of *Leishmania* [35].

**Table 3:** Classification of the infection status of the dogs. Classification system was used at each full clinical assessment and at the end of the study to determine the infection status of each dog.

Not infected (*Leishmania* free)	Non established infection	Active infection	
Not infected	Subpatent infection	Asymptomatic disease	Symptomatic disease
qPCR on bone marrow: Negative	qPCR on bone marrow: Positive (low burden)	qPCR on bone marrow: Positive	qPCR on bone marrow: Positive
qPCR on blood: Negative	qPCR on blood: Negative	qPCR on blood: Negative	qPCR on blood: Positive/Negative
Serological detection: Negative	Serological detection: Negative	Serological detection: Positive/Negative	**Serological detection: Positive/Negative**
No clinical signs	No clinical signs	No clinical signs	**Specific clinical signs (Leishmaniasis)**

*Subpatent infections* were defined as a non-established infection featuring transient or sustained detection of low quantities of parasite DNA in bone marrow from asymptomatic dogs with negative serological results. When the parasite burden was lower than the diagnostic threshold of the qPCR assay (27 < Ct < 40), it was considered as low quantity.

*Active infections* were defined as the parasitological detection in bone marrow. Therefore, a dog was classified as *actively infected* if its parasite burden in bone marrow was higher than the positive diagnostic threshold of the qPCR (Ct ≤ 27.0, equivalent to ≥0.18 parasites/mL of sample). Active infections were further sub-classified as *asymptomatic* and *symptomatic*. Symptomatic disease was defined by presence of a d.c.s. ≥ 8, composed by at least 3 or more clinical signs indicative of leishmaniasis (low body condition, onychogryphosis, muscular-cephalic atrophy, uveitis –grade ≥2, uni or bilateral-, lymphadenopathy –grade ≥2-, alopecia –grade ≥2-, exfoliative dermatitis –grade ≥2-, and ulcers –grade ≥2) and 2 or more biochemical deviations.

This status classification has to be interpreted as a flow in the disease development, from absence of infection to infected and clinically affected, showing potential intermediate steps. In addition, it has been proven that *subpatent infections* (qPCR positive animals with low parasite burden) might revert to not infected status whereas, as soon as the infection becomes active (qPCR positive with parasite burden higher than the diagnostic threshold), the dog’s immune system is generally no longer capable of fighting the active multiplication of the parasite, and the status shifts from asymptomatic to symptomatic. This progression is usually coupled with antibody increase and intermittent periods of parasitemia.

For an efficacy assessment, following previous recommendations [37], the percentages of subpatent, active infected, asymptomatic and symptomatic active infections were calculated by treatment group at days 28, 194, 374, 554, 644 and 734.

#### Determination of the degree of natural exposure to the parasite and prevalence.

Exposure to the parasite was calculated for each location and area based on serum data (two indirect ELISA) and or molecular data (qPCR on bone marrow) obtained during the exposure phase (Fig 1). Any animal with a positive score in any of the two ELISA tests (INgezim Leishmania or Civtest Canis Leishmania) and or a qPCR positive was considered as exposed according to the diagnostic criteria of the study. Exposure during the first year of the study after the primary vaccination phase reflected incidence, whereas prevalence was estimated as the exposure rate at the end of the study including seropositive and/or qPCR-positive dogs, where Ct ≤ 27 indicated active infection and 27 < Ct < 40 subpatent infection. Hence, prevalence was obtained from the cumulative number of positive control dogs in the parasitic diagnosis (serology and/or qPCR of bone marrow samples) detected at the end of the study (T734).

### Assessment of absence of interference of vaccine with IFAT test

The absence of interference with the Indirect Fluorescence Antibody Test (IFAT) was evaluated. IFAT assays were performed on serum samples obtained at days 28 (35 days after 2^nd^ dose), 208 (14 days after 3^rd^ dose), 388 (14 days after 4^th^ dose), 573 (14 days after 5^th^ dose), 644 (90 days after 5^th^ dose) and 748 p.v. (14 days after 6^th^ dose) from a random population of 15 vaccinated and 15 control dogs. IFAT assay was conducted by an external laboratory (Laboratory of Parasitology, Faculty of Veterinary Science, University of Zaragoza) using MegaFLUO LEISH (MegaCor Diagnostik GmbH, Austria) according to the reccommendations of the OIE Manual for Diagnosis Tests and Vaccines for Terrestrial Animals (https://sont.woah.org/portal/tool?le=en). For canine leishmaniasis, the positive threshold titer for IFAT test ranges between 1/40 (considered as doubtful result and indicative of exposure but not necessarily established infection) and 1/160 (indicative of established infection). When a titer value of 1/40 is detected, it is recommended to periodically follow up the suspected animal in order to confirm or discard the former result. A titer of 1/320 or higher may be indicative of the disease in clinically suspicious dogs [38].

### Assessment of product safety after each vaccination

After administering each vaccination dose, safety was assessed through individual observation of all animals enrolled in the trial, assessing the presence/absence of anaphylactic shock during 2 hours after vaccination and systemic signs (rectal temperature, anorexia, depression, behavioural changes, etc), and/or the presence or absence and severity of local reactions at the administration site (local lesions in nostrils –nasal discharge, nasal itching, sneezing, nasal edema and inflammation). The assessment was conducted by the trial investigators through daily visits for at least 4 d.p.v., as well as at 7 and 14 d.p.v. after each vaccination.

Vaccination of pregnant animals was not clearly defined in the inclusion criteria or considered as a factor for exclusion or withdrawal of the animal. On the other hand, as the study was conducted under natural conditions, there were females in all locations and some were at different stages of pregnancy when one or several vaccination doses were administered. This information was obtained a *posteriori*, i.e., each investigator was informed by the owner about the animal’s pregnancy status in the previous sampling. Given that the number of vaccinated pregnant females was significant, it was decided to document their vaccination properly, as well as safety follow-up on pregnancy and offspring.

### Statistical analysis

The established sample size was 361 dogs, of which 181 correspond to the vaccinated group and 180 to the control group. This sample size, considering withdrawal of 50% of the animals, makes it possible to detect a minimum efficacy of 57.5% (10% of estimated prevalence in all controls *versus* 4.25% in vaccinated dogs) with a confidence interval of 90% and a potency of 80%.

The minimum statistical unit was each study dog. The statistical data analysis was conducted for each area separately, as well as for the cumulative data for all the areas using the treatment group as a categorical variable (A or B). The null hypothesis (H_0_) was defined as the absence of significant differences between treatments, setting a significance level of α = 0.05, using two-tailed goodness-of-fit analyses for the statistical tests that allow it.

The percentage analysis (% infected, % symptomatic infected, etc.) was assessed using a 2x2 contingency table analysis (treatment group *vs*. affected/not affected) employing the Fisher’s exact test or the *Chi-square* test (df = 1), depending on the data structure. When significant differences were obtained, the risk ratio (RR, probability ratio) and the odds ratio (OR) of the factor were calculated, along with the 95% confidence interval (CI). The OR was calculated using the Odds Ratio Calculator programme (MedCalc for Windows, version 15.0, MedCalc Software, Ostend, Belgium). For the other continuous variables (clinical scores, parasite load in bone marrow and blood, serology data, cell response data, weights, temperatures, etc.), the treatment group was generally established as the categorical variable and parametric or non-parametric tests to compare means were used (variance analysis), depending on the results of the Kolmogorov-Smirnov & Lillefors and Shapiro-Wilk´s normality tests.

Following previous recommendations [37], the status of the vaccinated and control dogs was compared at the end of the study when efficacy of the vaccine was calculated using standard efficacy calculation ([%symptomatic controls - %symptomatic vaccinated/ %symptomatic controls]*100). Data from each dog withdrawn from the study were carried forward to the completion of the study for efficacy analysis, including parasite burden, parasitemia, and clinical signs. This statistical method is called *Last Observation Carried Forward* (LOCF) [37] and it is intended as a method that corrects potential bias caused by withdrawal.

## Results and discussion

Until now, five vaccines against canine leishmaniasis have been marketed. The second generation vaccines Leishmune and Leish-Tec were authorized in Brazil, but none is currently available. Leish-Tec consists of the *L. donovani* A2 amastigote recombinant antigen using saponin as the adjuvant [39], and Leishmune is made of a purified parasite glycoproteic fraction called fucose manose ligand (FML) antigen [40]. CaniLeish (*L. infantum* Excreted-Secreted Antigen (LiESP) plus QA21 adjuvant) and LetiFend were authorized by the EC based on the respective EMA opinions, but only the latter is available. Letifend consists of the chimeric protein Q, composed of five epitopes from four *L. infantum* proteins (LiP2a, LiP2b, LiP0, and histone H2A) and was tested against challenge with 5x10^5^ promastigotes per dog in preclinical phase [41], whereas Neoleish was tested against 10^8^. This chimeric protein had been previously evaluated for serodiagnosis, due to the high production of antibodies it induces, since it is a second generation vaccine [42]. In a double-blind randomized clinical field trial in which 549 dogs were enrolled, Fernández-Cotrina et al. [43] reported that Letifend efficacy is 72% in terms of clinical sign reduction in 2 selected kennels out of the 19 based on a higher disease incidence in the placebo group and the infection pressure in the area (reviewed by [15,44,45]). Further advances to control the burden of the disease are required (see the prevalence estimation calculated below). For this purpose, parasite burden control is required, specially in bone marrow, the main target tissue where the parasite persists. Not only diagnostic analysis of parasite presence is required to calculate the odds, but also quantification of the parasite burden in each infected animal. Neoleish is a DNA vaccine composed of the LACK gene included in the antibiotic resistance-free mammalian expression plasmid vector pPAL that significantly decreases parasite burden in bone marrow and clinical signs and activates a specific Th1 response [29,46,14]. Parasite detection was performed to evaluate exposure and infection rates in the previous canine leishmaniasis vaccines (reviewed by [15]) but only in Canileish [47] and Neoleish (see below) trials the parasite burden was quantified in bone marrow and blood. Neoleish was approved by the EC (December 2022) based on the EMA opinion (November, 2022) and included in the LeishVet guidelines (https://www.leishvet.org/wp-content/uploads/2025/07/LeishVet-Dog.pdf; [48,36]). The comparison of the five marketed vaccines and their status is summarized in Table 4.

**Table 4:** Marketed vaccines against canine leishmaniasis.

Trade name (generation/type)	Antigen/Adjuvant	Adminis-tration regimen	Protection (% clinical signs)		DIVA **	Quantification of parasite load (bone marrow)	Current marketing status
			No correction	LOCF* correction			
**Leishmune** (2^nd^/Purified glycoprotein fraction)	FML antigen	2 doses, annual booster	76	Not provided	No	No, only detection	Discontinued in 2014
**Leish-Tec** (2^nd^/Recombinant protein)	Recombinant A2 protein/ Saponin	3 doses, annual booster	71.4	Not provided	Yes	No, only detection	Discontinued in 2023
**CaniLeish** (2^nd^/Purified excreted/secreted (ESP) antigens)	LiESP/ QA21	3 doses, annual booster	Not provided	68.4	No	Yes	Discontinued in 2023
**Letifend** (2^nd^/Chimeric protein)	Q protein	1 dose, annual booster	72.0 (Results from 2 out of 19 kennels***)	Not provided	Yes	No, only detection	On the market
**Neoleish** (3^rd^/Naked plasmid DNA)	pPAL-LACK plasmid	2 doses, 6 months booster	72.7	64.5	Yes	Yes	On the market

*Last Observation Carried Forward. The LOCF method was applied when a dog was excluded from the study. The last result available was repeatedly reported until the end of the study. **DIVA. No interference with diagnosis. ***19 kennels were included in the study [43], but the 72% result corresponds to 2 selected kennels out of 19 ([43]; reviewed by [15,44,45]).

### Rate of exposure to *L. infantum* and prevalence

The *Phlebotomus* spp. vector seasons in Spain and Portugal generally span between the end of May and early October [37], and the estimated frequency distribution varies depending on the geographical location and the particular climate conditions of each year. However, the frequency generally peaks between July and September, as it was reported, even at high altitude locations in Sierra Nevada (Granada, Andalucía, Spain) between 2011 and 2013 [49]. The sandfly density studies are scarce, particularly for the 2017 and 2018 seasons. In certain areas of the Province of Girona (Catalonia, Spain), the exposure of dogs to the sand fly was generally comprised between 33% and 75% in July and September [50], and the sand fly season was confirmed to be during the summer, according to data taken in February, August, and October 2016, and January and April 2017 [51]. The *L. infantum* seroprevalence in dogs in a geographical area including the Provinces of Tarragona and Barcelona (Catalonia, Spain) was ~ 15.7%, and was comprised between 14.5 and 15.7% only considering the second. In a multicenter study of vectorial parasitic diseases in Northern Spain, Díaz-Regañón et al. [52] reported a general estimated *L. infantum* seroprevalence in dogs in Catalonia of 13.4% in 2017 and 2018, which are the years where dogs of this study were exposed to the vector. These data are similar to the *L. infantum* prevalence numbers obtained from a literature review for the 1985–2019 (14.5-16.7%) and new experimental data of the 2011–2016 periods combined with reviewed data (8–16%) concluding that the B area is an endemic zone with an intermediate-to-high risk [53].

In the cases of the Provinces of Cáceres and Badajoz (Extremadura, Spain), no direct sandfly dynamics and seroprevalence studies have been published. The *L. infantum* seroprevalence in the CA area varied between 14 and 34.2% between 1988 and 1995, whereas it was lower in the BA area (1.7-7%) according to data from 1997 and 2002–2003. In the 2011–2016 period, Gálvez et al. [53] estimated that the *L. infantum* seroprevalence in dogs was 19.8% in the CA area, and combined data for the 1985–2019 period led to the conclusion that the BA area is an endemic zone with intermediate-to-high risk and that the CA area is a hyperendemic zone with high *L. infantum* infection risk for dogs [53]. General data of *P. perniciosus* obtained in Spain between 2011 and 2013 showed a confluent biomodal distribution between June and September reaching the maximum frequency levels in August [54]. The same study reported that in Lisbon (Lisbon Region, Portugal), an area relatively close to the BA and CA areas, the distribution was also confluent bimodal peaking in June and September those years, and bimodal with peaks in June and September in the Algarve Region (Portugal). The Fuenlabrada outbreak focus (Autonomous Community of Madrid, Spain) is another area relatively close to these, although hares (*Lepus granatensis*) were described to be the main reservoirs instead of dogs [55,56]. González et al. [57] reported the peak of maximum captures in August in the 2012 season between June and September and less captures during 2013 and 2014 peaking between August and September in 2013 and 2014, a decrease presumably due to higher relative humidity over 50%, with an optimal value comprised between 30% and 40%. In summary, the presence of the vector in the areas of our field study is expected between the beginning of May and the end of September or the beginning of October, and peaks during the central months of the summer. This period began after the end of the primary vaccination phase and ended approximately when sampling at T194 p.v. was performed (Fig 1). Recruitment of unexposed dogs (*L. infantum* seronegative and qPCR-negative) was performed during January 2017, and the primary vaccination phase ended on April 4^th^, 2017; the first sample after primary vaccination was at T28 p.v. (14 days after the primary vaccination booster dose) (Fig 1). In this study, the exposure rate to *L. infantum* was calculated in the placebo-treated dogs by indirect ELISA (Ingezim/Civtest) and/or qPCR test (see below). Despite the unexposure gap between T194 and T374 p.v., the effects of exposure to the parasite were noticed during this period because more ELISA and/or qPCR positives (Ct < 40, where Ct ≤ 27 corresponds to active infection and 27 < Ct < 40 to subptatent infections) in bone marrow were detected. Overall, the parasite exposure rate of the control group during the first period was ~ 9%, considering the effects of exposure until T374 p.v. This exposure rate reflects incidence because only unexposed dogs were initially recruited. At the end of the study, parasite exposure of the control dog group was 22.78% (41/180), which reflects prevalence of *L. infantum* infection. Exposure of this group by study area was 5.08% (3/59), 21.31% (13/61), and 41.67% (25/60) in the BA, CC, and B areas, respectively. Notwithstanding, any dog with at least one positive result in any of the tests during the course of the study was considered exposed. After both exposure periods, the results showed that 106 dogs (53 vaccinated and 53 placebo-treated, respectively 29.2% and 29.4%) showed *L. infantum* exposure following the criteria detailed above, this including the cumulative data of *L. infantum* seropositives and/or qPCR-positives (active and subpatent infections). Exposure reached 15.3% in each group after the first sand fly season. Compared to the previously discussed epidemiological studies, the global transmission rate of the study was generally high, namely high in the B area, moderate-to-high in the CC area, and equal-to-low in the BA area. When a dog becomes infected, not always is this condition maintained until the end of the study, in agreement with previous works [37]. Some positive dogs become negative after time because infection is cleared, while infection progresses in some others, thus increasing the parasite load and the serological responses. The parasite burden, parasitemia, clinical outcome, and subpatent infection comparisons between the placebo and the vaccinated dogs are key indicators of Neoleish efficacy, as explained below.

### Initial demographic characteristics of the study population

**Fig 2:**
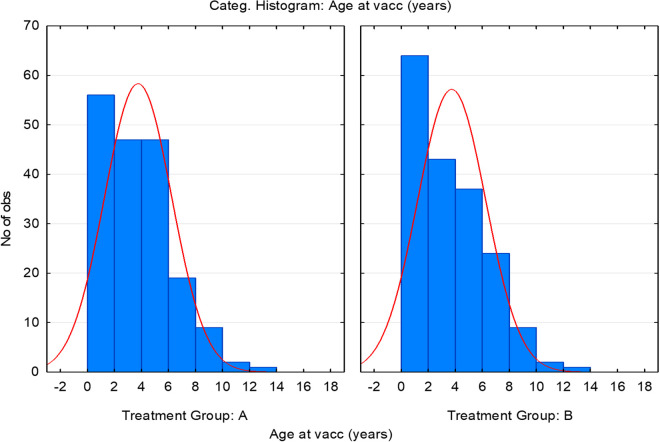
Categorical histogram (Nr. of observations) of age disclosed by treatment group at T0p.v. Group A: Vaccinated dogs. Group B: Placebo dogs.

The age of the total population enrolled (n = 361) ranged from 6.8 months to 16.10 years. The mean age of the dogs was 3.81 years ± 2.54 (mean±SD). 95% CI% of the mean was 3.56-4.05, lower and upper quartiles = 1.80 and 5.48 respectively. The mean age of vaccinated animals was 3.72 ± 2.47 (n vac = 181) and 3.69 ± 2.51 in the control animals (n cont = 180) with no statistically significant differences between both groups (Fig 2).

In the vaccinated group 57.46% (104/181) were males and 42.54% (77/181) were females, whereas in the placebo group 58.89% (106/180) were males and 41.11% (74/180) were females, with no statistically significant differences in the sex distribution between both treatment groups. 192 different pure breeds (95 vaccinated and 96 controls) and 169 crossbreed dogs (85 vaccinated and 84 placebo) were included in the study. Pure breeds involved were: Alano, Beagle, Boxer, Bracco, Drahthaar, Great Gascony Blue, Spanish Mastiff, Majorca Shepherd, Podenco (Andalusian, Canario, Ibérico and Portuguese), Spanish Hound and English Setter. The most frequent crossbreed was a mix of Spanish Podenco and Mastiff. No significant difference between both groups was observed in breed distribution. As a conclusion of the demographic data distribution, the study was well-balanced and no bias caused by the population structure was expected.

### Assessment of the number of seropositive dogs against crude *Leishmania* antigen (CLA) measured by ELISA and absence of interference with diagnosis

All serum samples were tested in duplicate using the INgezim *Leishmania* kit and those samples with doubtful or positive results were re-tested with the Civtest kit as a confirmatory assay. The rationale of this two-steps diagnostic procedure was as follows. Both indirect ELISA tests use crude immunodominant *L. infantum* antigens for capturing specific IgGs. For detection, the INgezim test uses conjugation to a specific canine IgG monoclonal antibody, whilst Civtest uses a generic protein A/HRPO conjugate. The validity of these tests has been assessed by the manufacturers using IFAT as the reference test [58,59]. The Ingezim kit showed 95% and 80% agreement for 1/100 and 1/160 IFAT cut-offs positive values, respectively, whilst the estimated sensitivity and specificity of the Civtest assay were 98% and 96% respectively. Moreover, the performance of both tests was recently assessed on experimentally infected dogs [60], and specificity was 100% for both tests whilst sensitivity was 98% for Civtest and 78% for Ingezim. Therefore, Civtest was used as a second-line diagnostic in order to confirm the first Ingezim ELISA result and to increase the specificity of the serological assessment.

**Fig 3:**
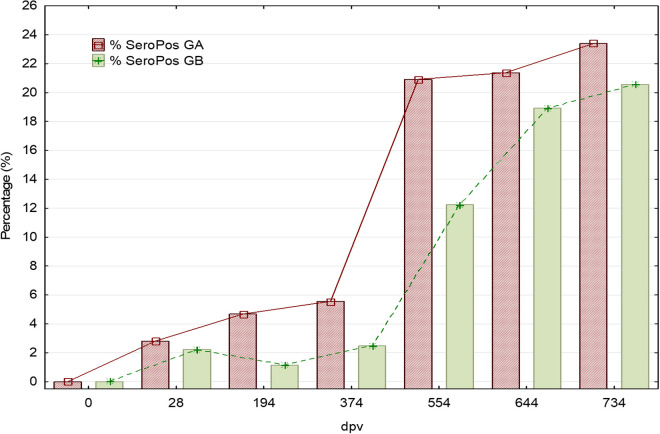
Progression of the percentage (%) of seropositive dogs detected by indirect ELISA (Ingezim test) in vaccinated and placebo-treated dogs. Vaccinations were performed at days: 0, 14, 194, 374, 554, and 734. Additional samplings were performed on day 28 (14 dpv after 2^nd^ dose) and 644 (90 days after 5^th^ dose). GA: Vaccinated group. GB: Placebo group.

Fig 3 shows the number and proportion of dogs (*vs*. total number of dogs available per group) with positive serological response in both groups at each study time point. The number of dogs that became seropositive increased gradually during the study in both groups, and the number of dogs that seroconverted was similar in both groups at most of the time points, with no statistical differences in the percentages. At the last time point (T734 p.v.) 23.40% (33/141) of the vaccinated and 20.57% (29/141) of the control group were seropositive. The proportion of seropositive vaccinated dogs is very low but tends to be slightly higher throughout the time points studied before day 554 p.v., which indicates the absence of interference with serological diagnosis test. From day 554 p.v. on, a prominent increase is observed and maintained over time. As reflected in the following sections, this time point marked a break in the progression of the disease course during the time period studied. The rate of seropositive dogs reported in a double-blind randomized field clinical trial of Letifend was 9.7% (18/186) and 7.0% (12/171) at the endopoint (730 p.v.) [43]. In a randomized controlled trial of Canileish under field conditions, 29.6% of the positive dogs for anti-*L. infantum* antibodies were vaccinated and 18.9% from the control group one year after vaccination [47], which are comparable to the antibody levels obtained with Neoleish after natural infection.

### Parasitological follow-up and parasite burden

#### Bone marrow.

qPCR in bone marrow samples is one of the most sensitive and specific approaches for diagnosis of leishmaniasis [35] due to the fact that this is the most relevant lymphoid tissue for parasites replication. Parasite detection bone marrow is indicative of active infection, and when high levels are found in this tissue, parasite in blood are intermittently found (parasitemia) with the highest probability of transmission [61,35]. Only a proportion of animals with high levels of parasite burden in target replication tissues, mainly bone marrow and spleen, show intermittent cycles of parasitemia [62]. On days 0, 28, 194, 374, 554, 644 and 734 d.p.v., parasite detection and quantification was performed by qPCR on bone marrow samples. Parasite load was additionally measured in blood samples in those animals with a positive value (Ct < 40; see below).

**Fig 4:**
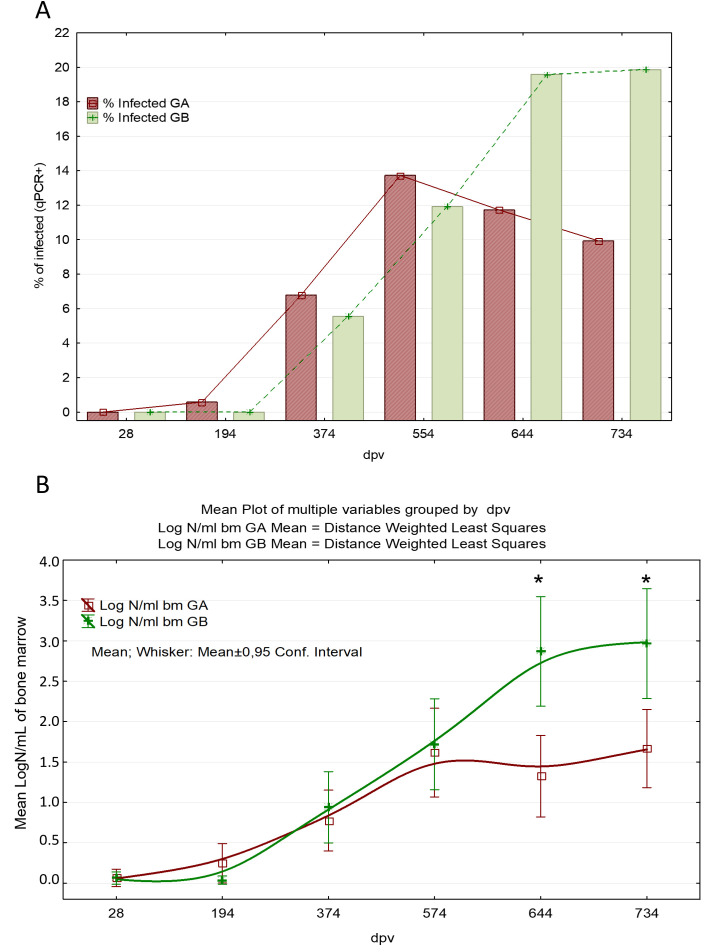
Detection and quantification of *L. infantum* in bone marrow by qPCR. **(A)** Proportion (%) of active infected dogs (presence of *Leishmania* in bone marrow with Ct ≤ 27.0) throughout the study period (*p < 0.05, Fisher’s exact test). Group A (GA): Vaccinated; Group (GB): Control. **(B)** Arithmetic means of the bone marrow parasite load over the course of the study in qPCR positive dogs (Ct < 40). Plotted curves and distance to mean values are weighted by least squares. Vaccinations were performed at days 0, 14, 194, 374, 554 and 734 p.v. Additional samplings were conducted at days 28 and 644 p.v. GA: Vaccinated; GB: Control. LOCF method was used for missing values. (* p < 0.05 vaccinated *vs*. placebo).

Prevalence was evaluated exclusively according to qPCR results in bone marrow at each time point, regardless of the serological status detected by ELISA. The total number and percentage of dogs with presence of *L. infantum* parasite in the target replication site progressively increased in both treatment groups over the course of the study, and the percentage of infected animals was statistically significant lower in the vaccinated group on days 644 and 734 p.v. when compared with the placebo group (Fig 4A). The observed tendency in the rate of infected dogs correlates to the progression of parasite burden in bone marrow and blood, with a similar breaking point at 554 d.p.v. On day 734 p.v. the presence of *L. infantum* infection was demonstrated in 9.93% (14/141) of vaccinated and 19.86% (28/141) of control dogs (probability or risk ratio, RR = 2; odds-ratio, OR=2.24), and this difference is statistically significant (p = 0.031). Hence, control dogs have a probability 2 times higher (RR = 2) to be actively infected than vaccinated and the significant effect of vaccination in the reduction of the percentage of animals actively infected was shown. The rates for Letifend were 9.4% (30/186) for the vaccinated group and 16.1% for the placebo (16/171) according to PCR or smear test in lymph node or bone marrow at 730 p.v. [43]. As for Leishmune, 25% of the vaccinated and 75% of the control were qPCR positive in the same tissue according to Giemsa-stained tissue smears and cultures from the bone marrow samples [63]. A randomized controlled trial of CaniLeish under field conditions revealed that 42.9% of the serologically positive animals were qPCR positive in lymph nodes in the vaccinated group and 71% in the control group (reviewed by [47]. However, the mere analysis of parasite presence does not discriminate subptatent infections from infection progression. Parasite burden quantification is a better indicator of efficacy. qPCR analysis for Neoleish revealed a mean parasite burden (S1 Table) significantly lower (~96.8 and ~95.5%) in the vaccinated group on days 644 and 734 p.v. than in the control group (Fig 4B). In addition, it should be noticed that the mean parasite load in vaccinated animals was also lower on days 374 and 554 p.v.

The results obtained in the specific cohort of PCR positive animals (Ct value <40 at any time point) showed statistically lower parasite burden in those vaccinated than in the placebo group on days 644 and 734 p.v. For the statistical analyses, the LOCF method was applied following previous recommendations [37]. Hence, when a dog was withdrawn, the last result available was repeatedly reported until the end of the trial.

Infection progression in natural conditions has variable latency periods approximately ranging from 6 to 18 months, with low parasite replication and absence of disease manifestations. Therefore, it can be concluded on the basis of parasite burden results (Fig 4) that vaccination affected the progression of parasite multiplication in the replication target tissue showing a significant reduction of parasite load in vaccinated animals.

#### Blood. Proportion of parasitemic dogs.

**Fig 5:**
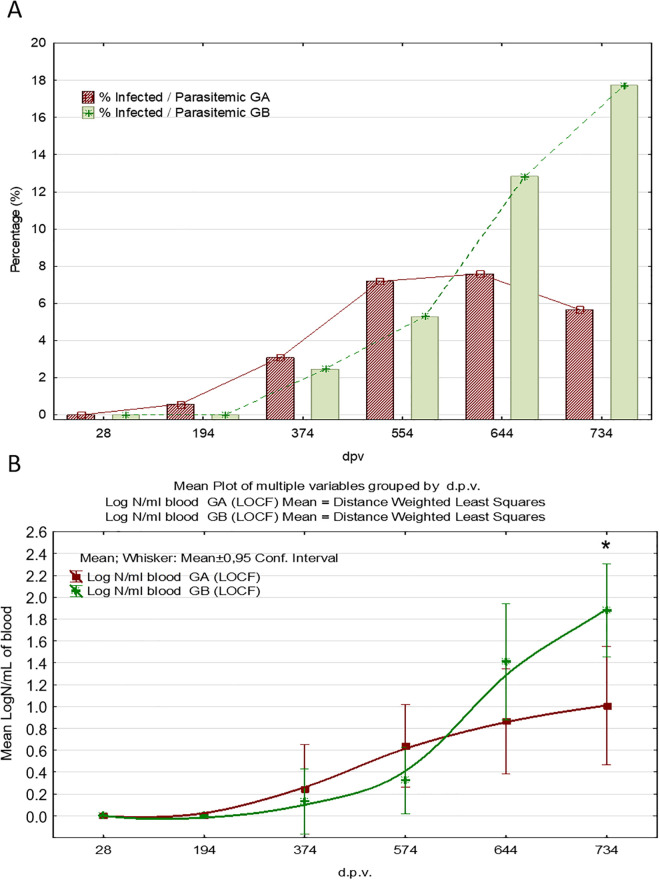
Detection and quantification of *L. infantum* in blood. **(A)** Proportion (%) of infected dogs (vaccinated and control) with progression to parasitemia throughout the study period (*p < 0.05, Fisher’s exact test). GA: Vaccinated; GB: Control. **(B)**Means of the blood parasite load over the course of the study in qPCR positive dogs (Ct < 40). Plotted curves and distance to mean values are weighted by least squares. GA: Vaccinated, N_vac_ = 13; GB: Control, N_cont_ = 23 (* p<0.05 vaccinated *vs*. placebo-treated). The LOCF method was used for missing values.

Parasite detection and quantification in blood for those animals with positive molecular detection in bone marrow was performed by real-time qPCR on days 0, 28, 194, 374, 554, 644 and 734 p.v. The LOCF method was applied for correction of bias caused by animal withdrawal during the trial. In addition, samples with negative qPCR results were transformed to a 0 value. Vaccinated and control groups displayed an upward trend in the percentage of parasitemic dogs (i.e., presence of *L. infantum* in peripheral blood determined by qPCR) until T554, and a declining progression in the vaccinated dogs between days 644 and 734 p.v. (Fig 5A). At the end of the study, the proportion of infected dogs that presented *Leishmania* in blood was 5.67% and 17.73% for the vaccinated and the control group (OR = 3.5), respectively. This difference was statistically significant (Fisher ’s exact test). The statistical significance was similar when missing values were included.

The mean parasite burden in blood (parasitemia), only including in this analysis dogs with at least one positive result (mean Ct < 40.0) at any time point (S2 Table) was significantly lower (86%) in the vaccinated than in the placebo group at T734p.v. (Fig 5B). In addition, it should be noticed that the mean parasite load in vaccinated animals was also lower on day 644 p.v. No data on the rate of parasitemic dogs and quantification of parasitemia have been reported for any vaccine except for Leishmune and Neoleish. 5.1% vs. 36.6% of the Leishmune-vaccinated vs. control dogs were infectious to sandflies according to xenodiagnostic analysis, respectively [64].

Parasite burden was lower in blood than in bone marrow, but it should be highlighted that bone marrow is one of the target tissues the parasite shows tropism for replication and persistence, and only a low proportion of amastigotes escape from replication sites to the circulatory system.

### Clinical assessment and progression of *Leishmania* infection to clinical disease

The frequency of clinical evaluations is based on the previous experience with the Neoleish vaccine [29] and other recommendations [65,66] showing that the clinical development has an approximated latency period of 6 months to 1 year after natural infection. Table 5 shows the mean daily clinical scores assessed on days 0, 28, 194, 240, 314, 374, 450, 554, 644 and 734 p.v

**Table 5:** Progression of mean clinical scores (d.c.s. and g.c.s.). The Last Observation Carried Forward (LOCF) method was used to correct bias caused by missing values of withdrawn dogs. (*p < 0.05).

dpv	Mean d.c.s. Vaccinated	Mean d.c.s Placebo	U-value	Z	p-value	Valid N Vacc.	Valid N Placebo	Std.Dev. Vacc.	Std.Dev. Placebo	Mean Vac (- Mean Placebo)	Confidence (-95.0%)	Confidence (+95.0%)
**dcs T0pv**	0.728	0.911	15181.50	-0.94390	0.345223	181	180	1.302	1.533	1.386	0.030	-0.183
**dcs T28pv**	0.722	0.687	16086.50	0.02339	0.981336	181	180	1.291	1.205	1.147	0.361	0.035
**dcs T194pv**	0.956	0.899	15932.00	-0.18054	0.856728	181	180	1.437	1.294	1.233	0.163	0.056
**dcs T240pv**	0.972	0.994	15307.50	-0.81574	0.414650	181	180	1.432	1.313	1.188	0.250	-0.022
**dcs T314pv**	0.706	1.296	12321.00	-3.85340	0.000117*	181	180	0.996	1.486	2.230	0.000	-0.591
**dcs T374pv**	1.094	1.117	15770.50	-0.34481	0.730240	181	180	1.417	1.411	1.008	0.958	-0.023
**dcs T450pv**	0.917	1.654	12309.00	-3.86560	0.000111*	181	180	1.082	1.778	2.697	0.000	-0.737
**dcs T554pv**	1.456	1.654	14477.50	-1.65996	0.096924	181	180	1.901	1.831	1.078	0.617	-0.198
**dcs T644pv**	1.733	1.804	15171.50	-0.95407	0.340050	181	180	2.040	1.833	1.239	0.154	-0.071
**dcs T734pv**	1.583	1.715	14557.50	-1.57859	0.114432	181	180	2.156	1,903	1,283	0.097	-0,132
**gcs**	10.747	12.732	14161.50	-2.14556	0.031908*	181	180	11.244	11.765	1.095	0.545	-1.985

In the control group, clinical manifestations appeared around day 314 p.v. and progressively worsened until the end of the study. Vaccinated dogs showed a significant delay in the development of clinical signs, with a progressive increase between days 554 and 644, and dropping on day 734 p.v. (Table 5).

**Fig 6:**
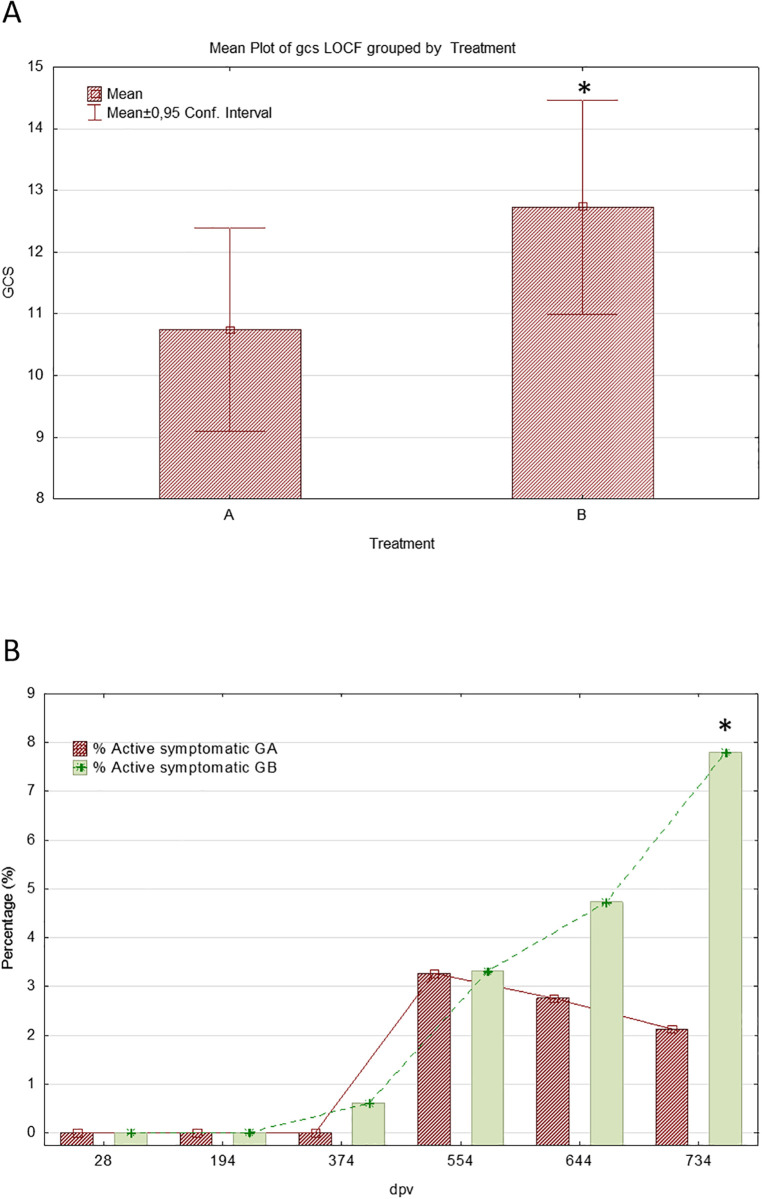
Clinical signs. **(A)** Comparison of mean accumulated clinical scores (g.c.s.). GA: Vaccinated; GB: Control. (*p < 0.05). **(B)** Proportion (%) of vaccinated and control dogs that progressed to leishmaniasis (symptomatic cases of disease) throughout the study period. *p < 0.05, Fisher’s exact test. GA: Vaccinated; GB: Control.

Daily clinical scores were significantly higher in the control group on days 314 and 450, indicating at these points a lower severity of clinical signs in the vaccinated dogs. In addition, global clinical scores (g.c.s.) showed a statistically significant lower value in vaccinated dogs than in treated-placebo animals, indicating an overall lower severity of the development of clinical manifestations in vaccinated animals (Fig 6A). Both parameters (d.c.s. and g.c.s.) demonstrated statistically significant lower progression in the clinical development of the vaccinated animals.

Finally, clinical development presented a parallel progression with parasite burden in bone marrow and peripheral blood. As parasite burden progressively increased during the course of the study, clinical scores gradually increased. Therefore, peak values of parasite burden correlated with the higher severity scores.

It is important to note that infection is not equivalent to disease in the case of *Leishmania* [35]. Therefore, the high variability and complexity of clinical development implies that a confirmed case of leishmaniasis should be considered whenever: *(i)* The animal shows several compatible clinical signs of the disease (including alterations in biochemical parameters); and *(ii)* A positive parasite detection result in bone marrow is confirmed. Therefore, vaccine candidates should protect against both: infection rate and clinical development in infected dogs.

In this study, it was considered that an infected dog becomes symptomatic when it has a clinical score ≥ 8. This includes in such score, the sum of at least 3 or more clinical specific signs and 2 or more biochemical alterations, both at the end of the study (T734 p.v.) or at the last time point available before withdrawal. The clinical signs observed in infected dogs that developed clinical leishmaniasis are detailed in S3 Table.

In addition, a summary of the number and percentage of *active infected* animals (PCR positives with Ct ≤ 27) that progressed to *symptomatic* (clinical disease) or without disease progression (*asymptomatic*) to the end of the study is shown in S4 Table. LOCF has been included as an additional cohort including accumulated missing values until T734. The percentage of symptomatic vaccinated and control animals increased progressively over the course of the study with a parallel progression until T554 p.v. At this time point both groups showed different figures, with an increase in the control group and a gradual decline in the vaccinated (Fig 6B; S4 Table). At the end of the study (T734), the proportion of vaccinated dogs that became symptomatic was lower than placebo-treated animals (2.13% of vaccinated (3/141) *vs*. 7.80% of controls (11/141)) (Fig 6B). This difference was statiscally significant (Fisher’s exact test). Therefore, the odds-ratio is approximately 3.9 (see below), although the LOCF-corrected value is 3. In the case of Letifend, the rates of dogs that became symptomatic was also higher in the placebo (10.2%, 19/186) than in the vaccinated group (4.7%, 8/171) [43] (LOCF-uncorrected data). At the end of the randomized controlled field trial of Canileish, 87.6% were asymptomatic (87.3% of vaccinated and 87.8% of the control dogs [47]. Clinical signs were assessed in the largest Leish-Tec study and for Leishmune and no significant differences were observed between vaccinated and control groups (reviewed [15]).

**Fig 7:**
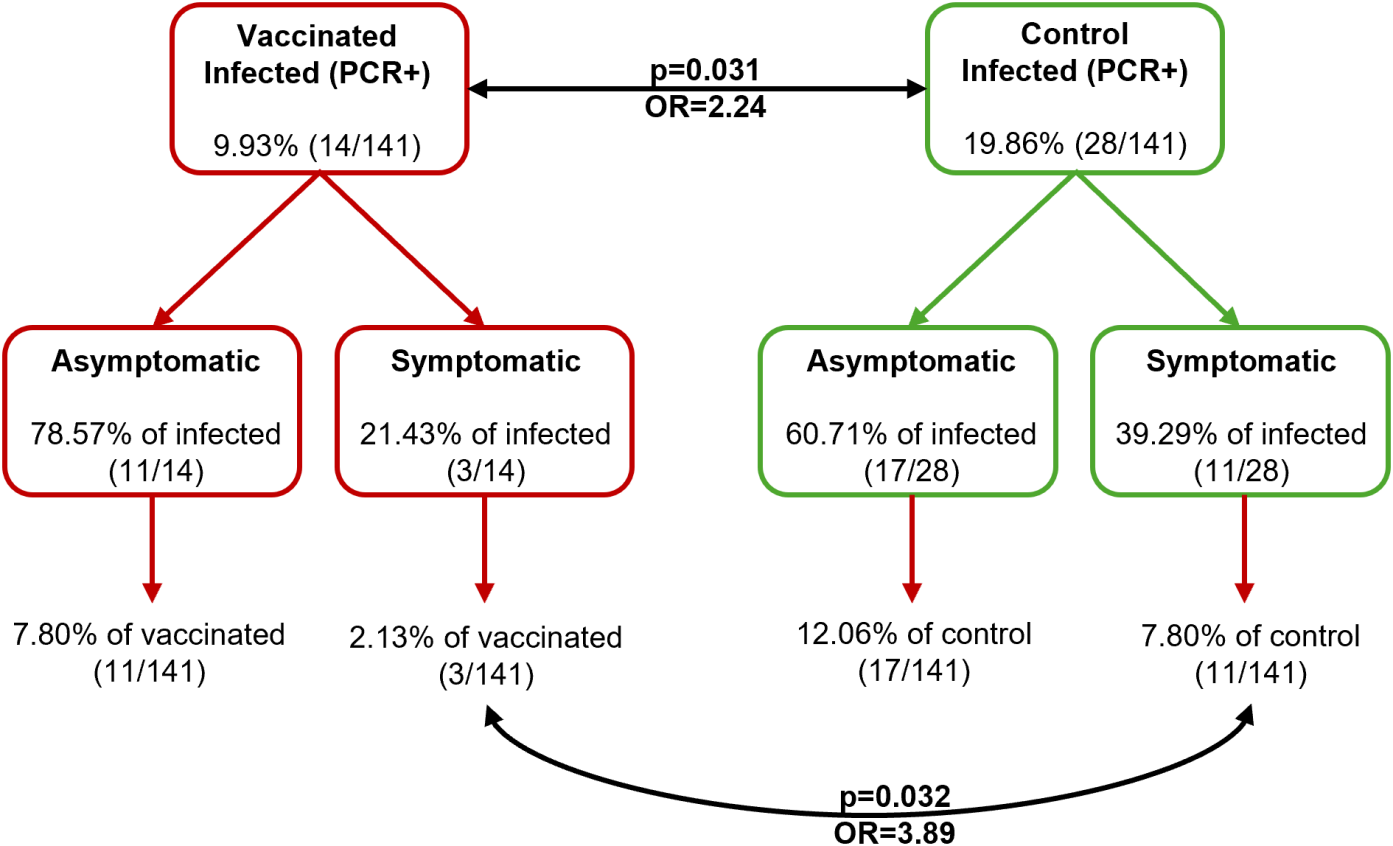
Summary of the dogs’ *Leishmania* infection status at day 734 p.v. after two years of exposure in an endemic area with active transmission.

Fig 7 summarizes the proportion of infected dogs in each treatment group at the end of the study, the proportion of infected vaccinated and control animals that were symptomatic/asymptomatic *vs*. the number of those infected within each treatment group, and the proportion of infected symptomatic/asymptomatic compared to the total population available in each treatment group at the end of the study. 21.43% of infected vaccinated dogs progressed to symptomatic, and this proportion was 39.29% in the infected controls. Furthermore, the proportion of infected control dogs that remained asymptomatic (60.71%) was lower than the proportion in infected vaccinated (78.57%). Due to the low number of animals infected, these proportions were not significant. However it is remarkable that the percentage of symptomatic vaccinated was approximately half of the symptomatic controls (21.43% in vaccinated *vs*. 39.29% in controls).

The overall efficacy of Neoleish in the prevention of progression to uncontrolled active infection at the end of the study was 72.7% (2.13% of vaccinated *vs*. 7.80% of controls; Fig 7; Table 4). These results are similar to those described for Fernández-Cotrina et al. [43] for the second generation vaccine Letifend in 2 out of 19 kennels (Table 4; reviewed by [15,44,45]), but both were calculated without applying the LOCF correction recommended by Oliva et al. [37]. According to meta-analysis by Calzetta et al. [67], Letifend efficacy is actually lower (reviewed by [68]). The calculation for Neoleish applying the LOCF correction is 64.52% (2.76% of vaccinated (5/181) *vs*. 7.78% of controls (14/180); Tables 4 and S4), with a LOCF-corrected odds ratio of 3. Therefore, the likelihood that a dog vaccinated with Neoleish would develop clinical signs of the disease is 3.89 times lower (3 after LOCF correction) than for an unvaccinated dog.

### Assessment of proportion of non-established infections and non-infected dogs

**Fig 8:**
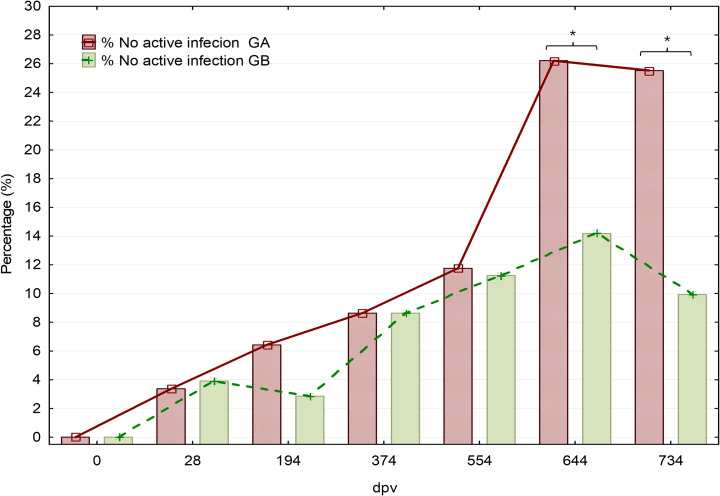
Progression of the proportion (%) of subpatent infections in vaccinated and control groups, over the course of the trial. *p < 0.05, Fisher’s exact test. GA: Vaccinated. GB: Control.

The main definition of *subpatent infection* (or *not established infection*) was based on the molecular detection of parasites in lower quantities than the positive threshold of the assay (<0.18 parasites/mL of sample, at Ct ≤ 27.0). S5 Table details the total number and proportion of subpatent infections, displayed according to serological status and/or the molecular detection of the parasite in quantities lower than the positive threshold of the assay. This table also shows the number and percentages of animals free of *Leishmania* (negative in all diagnostic tests). It should be noticed that the proportion of subpatent infections uniquely detected by qPCR progressively augmented in both groups, showing higher increase in vaccinated dogs; this difference was statistically significant in the last two samplings (T644 and 734, Fig 8). At the end of the study, the percentage of subpatent infections detected by qPCR was 17.02% (24/141) in the vaccinated group and 4.26% (6/141) in the placebo-treated. This effect is maintained if missing values are included in the analysis (LOCF). The overall protection rate of Neoleish, calculated as previously recommended [37], and according to the percentage of non-symptomatic vaccinated animals, was 70.17% (127/181).

**Fig 9:**
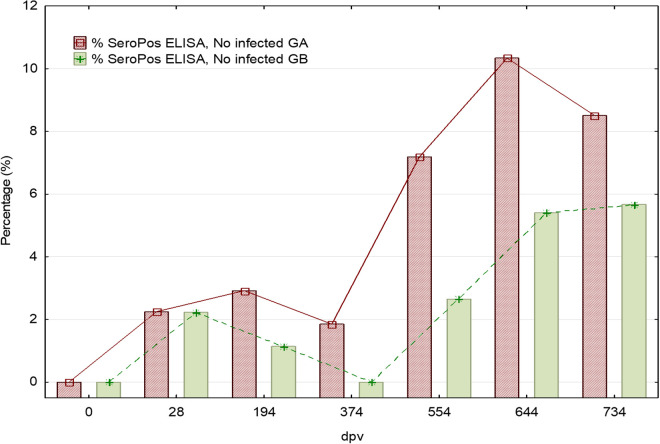
Progression of the percentage (%) of non-infected (PCR negative) seropositive. GA: Vaccinated. GB: Control.

The higher proportion of subpatent infections could be related or even caused by the significantly lower proportion of infected and the higher proportion of asymptomatic dogs observed in the vaccinated group, and it could be indicative of an immunological effect that leads to delay or even the disruption of the disease progression, increasing the number of subpatent infections and reducing the number of actively infected animals. This phenomenon could be similarly related with the potential spontaneous reversion to PCR negative status in dogs previously identified as actively infected (see above). Furthermore, the progression of the proportion of non-infected dogs (negative to PCR) that became seropositive in the vaccinated group, in comparison with the control, was also assayed. The proportion of vaccinated dogs that became seropositive tend to be higher than in placebo (Fig 9). This effect may be related with the effect of serological responses to repeated vaccine doses and non-infective or infective natural contacts with *Leishmania*.

### Assessment of the lack of interference of the vaccine against the IFAT serological test

IFAT results generally agree with all other diagnostic assays. As showed in S6 Table, in the vaccinated group, all IFAT positive dogs (IFAT titer >1/40) were, at least, positives to qPCR of bone marrow samples, showing Ct values lower or close to the positive diagnostic threshold of the molecular test (Ct ≤ 27). In addition, molecular diagnostic was usually confirmed in subsequent samplings by ELISA tests.

Two cases (dogs Id. 8712270197 and 8711137439, from vaccinated group) showed two repeated doubtful IFAT values (titer 1/40) on days 208 and 573, with an additional doubtful result on day 644 for the dog 8711137439. In addition, a 1/40 titer was detected by IFAT on day 644 for dog Id. 8712392896 (negative for the rest of techniques and for IFAT in all other samplings). Finally, the dog id. 941000015294084 with a doubtful result for IFAT on day 388 was confirmed as potentially infected by qPCR (Ct lower than the positive threshold) in the subsequent samplings.

In conclusion, repeated vaccination with Neoleish did not interfere with the reference laboratory test (IFAT) as none of the vaccinated non-infected dogs displayed titers higher than the positive threshold of the assay (1/40).

Letifend does not interfere with diagnosis [ ], as well as Neoleish. Leish-Tec induces positive anti-A2 antibody titers one month after vaccination in 98% vaccinated dogs [69] and interferes with diagnosis [64]. However, a vaccine inducing high antibody titers against the inoculated immunogen does not exclude the possibility that the vaccine interferes with diagnosis methods. For example, Leishmune induces anti-FML antibodies in 100% of the vaccinated dogs [63] but the interference outcome depends on the serological test [70]. Canileish interferes with diagnosis [71].

### Local and systemic safety

The local and systemic safety assessment was conducted for all animals available during each vaccination and re-vaccination, excluding animals that were withdrawn from the study. Therefore, the presence of shock and systemic signs such as anorexia, depression, behavioural changes, etc. was assessed in all animals. Adverse local reactions on the administration site were also assessed, such as: local lesions in nostrils, nasal discharge, nose itch, sneezing, nasal oedema and inflammation.

After administration of the primary vaccination regime (2 doses separated 14 days), as well as after administration of booster doses, no animals showed symptoms of anaphylactic shock during the 2 hours after vaccination for any of the doses administered or any other local or systemic disorder, either serious or mild. Given the cynegetic aptitude of the animals included in the study, it was particularly useful to verify the lack of changes in the sense of smell or any other local disorder derived from the intranasal vaccination.

No serious or mild systemic reactions were observed. No behavioural changes, such as anorexia or depression, or behavioural changes characteristic of the breeds during hunting activities, were observed. No plasmid integration was detected in the dog genome, and quantitative risk assessment considering the worst hypothetical scenario found the integration risk to be negligible [16]. Therefore, the repeated administration of Neoleish doses did not cause local or systemic adverse effects of any kind (either serious or non-serious), confirming the high safety profile detected in laboratory tests.

Leish-Tec general adverse reactions were observed, more often after the second or third vaccine dose in 11% of the cases [39]. Leishmnue showed a better safety profile [72]. In the case of Canileish, swelling, nodule haredning and other moderate and temporary reactions were observed to appear and resolve in up to 2 weeks after injection in some dogs [73]. Lethargy, vomiting, diarrhoea, and hyperthermia were observed after vaccination with Letifend in rare cases [74]. Hence, it can be concluded that Neoleish has the highest safety profile, not showing any alteration.

### Safety assessment in pregnant females

During the study, 32 pregnant females were detected, for which full information was obtained on gestation status at the moment of vaccination, gestation progress, due date and litter size in 18 cases (N_vac_ = 7, N_cont_ = 11). On the other hand, 3 pregnant females (2 from the vaccinated group and 1 from the control group) received 2 treatment doses in subsequent gestations (S7 Table).

The result was the treatment of pregnant females during the study, including the number of animals per treatment group, gestation status at the time of treatment (first and last third) and litter sizes. Considering the three animals with repeated doses as independent animals, there were data for a total of 21 pregnancies that were treated during the study, of which 12 were in control females and 9 in vaccinated females. In 6 cases, vaccinated animals were treated during the first third of the pregnancy (days 1–22) and in 15 cases females were treated during the last third (45–65 days of gestation). No effects on gestation or offspring, abortigenic effects or teratogenic effects were observed in any of the treated animals, vaccinated or controls, regardless of the gestation stage. There were no statistically significant differences in mean litter size (Mann-Whitney U Test, p = 0.38, 2-sided exact p) for all vaccinated and control females (N = 21, Nvac = 9, Ncontr = 12), with a mean size of 8.0 pups/litter in vaccinated dogs and 7.6 in those treated with placebo (S8 Table).

### Intranasal route administration and possible immunization mechanism

Many studies have been performed to prove the efficiency of intranasal vaccination, including some that underwent clinical trials in both respiratory and systemic infections. Intranasal vaccines can activate the immune system cells found on the upper and lower respiratory tract mucosa. This dual stimulation of the immune system, along with non-invasive administration, favors the development of novel nasal vaccines to produce long-lasting immunity [75]. The levels of protection induced are higher when administered by nebulization [76], as chosen for Neoleish. Selection of a method to improve DNA internalization into eukaryotic cells is required for DNA vaccines to obtain acceptable efficacies. The intranasal nebulization method was chosen for Neoleish for two reasons: i) To avoid complex and more invasive methods such as electroporation or needle-free systems; ii) Because abundant antigen-presenting cells can be found in the nasal mucosa, as well as TLR-9 receptors, which are able to recognise CpG motifs. The *fab*I gene included in the pPAL plasmid [28] contains a long CpG sequence.

To date, four different nasal vaccines have been approved by the regulatory authorities for human use against influenza A and B, and hepatitis in the USA, Europe, Asia, and Cuba markets. FluMist is a quadrivalent vaccine approved by the FDA and ensures immunization against the influenza A and B subtypes. Accordingly, Nasovac S is a trivalent nasal flu vaccine marketed in Asia. In 2016, the pandemic influenza vaccine Η5Ν1, formulated in suspension, was licensed in Europe for the prevention against a single strain of influenza virus. In addition, HeberNasvac is the only approved nasal vaccine, licensed in Cuba, for immunization against hepatitis B [75].

The mechanism of action for DNA vaccines generally unfolds across three essential stages: uptake, antigen production and processing, and antigen presentation [77]. Three primary mechanisms have been suggested for the cellular uptake of the plasmid DNA: 1) Direct passage through pores in the plasma membrane; 2) Micropinocytosis, which includes internalization via receptor-mediated or caveolar endocytosis; or 3) Endocytosis of extracellular molecules that have bound the plasmid, internalizing them through invaginations of the plasma membrane. Once inside the host cell, the plasmid DNA is transported to the nucleus for transcription, utilizing the host cell’s own protein expression machinery to produce the target antigens. These translated antigens are subsequently processed, leading to the presentation of peptides via MHC-I or MHC-II molecules. The products encoded by DNA vaccines thus possess the capacity to trigger the activation of both the innate and adaptive immune response. Furthermore, DNA vaccines activate the innate immune response through multiple mechanisms, primarily due to the recognition of CpG motifs by TLR9 receptors [77].

The mechanism of action for the Neoleish vaccine presumably follows this established route for DNA vaccines. The pPAL plasmid used in Neoleish contains a long CpG sequence, which presumably functions as an intramolecular adjuvant by engaging the innate immune system. Specifically, the recognition of these unmethylated CpG dinucleotides by TLR9 in endosomes of immune cells, such as dendritic cells, triggers the production of pro-inflammatory cytokines like IL-12, a key factor in driving the Th1 response. The LACK antigen contains peptide sequences that can bind sequences of vacuolar proteins involved in DNA replication and RNA synthesis. This suggests the involvement of these LACK determinants in the host response against the parasite through a hypothetical MHC-II-binding ability, possibly triggering cytokine production, [17]. The nebulization delivery method is highly advantageous, offering a friendly administration route that avoids volume losses and may significantly enhance the *in vivo* transfection efficiency of the plasmid into target cells. This efficiency potentially allows for the use of lower vaccine doses while still inducing higher levels of protection [76]. The nasal mucosa is particularly rich in TLR9 receptors, which readily recognize the CpG motifs present in the plasmid. Following antigen production, antigen-presenting cells process and present the LACK peptides primarily via MHC-II to naïve T cells. This presentation via MHC-II likely occurs when antigen-presenting cells, such as dendritic cells, phagocytose apoptotic or necrotic host cells that have expressed the LACK antigen, routing the exogenous antigen into the MHC-II pathway. Direct transfection of antigen-presenting cells may also occur. For Neoleish, this presentation is critical for polarizing the adaptive immune response toward a Th1 profile [29], which is essential for effective defense against intracellular pathogens like *Leishmania*. The Th1 response is characterized by the secretion of IFN-γ, which activate macrophages to kill the intracellular parasites, crucial for resolving leishmaniasis.

## Conclusions

Neoleish has shown to be safe and efficacious in previous laboratory studies and its safety and efficacy profile has been confirmed in this GCP-compliant field trial. First, vaccination dramatically affected the progression of parasite multiplication in bone marrow and peripheral blood showing a statistically significant reduction of parasite load in vaccinated animals at the end of the study. Second, the presence of *Leishmania* active infection at the end of the study was demonstrated in 9.93% of vaccinated and 19.86% of control dogs (RR = 2; OR=2.24), and this difference was statistically significant. Third, daily clinical scores (d.c.s) in the control group were significantly higher on days 314 and 450 indicating a lower severity of clinical signs in the vaccinated group at these time points. In addition, accumulated clinical scores (g.c.s.) showed statistically significant lower values in vaccinated dogs, indicating an overall lower severity on the development of clinical manifestations. Consequently, both parameters (d.c.s and g.c.s.) demonstrated a statistically significant lower progression rate of clinical development in the vaccinated dogs. Moreover, at the end of the study (T734), the proportion of infected dogs that became symptomatic was higher in placebo than in vaccinated dogs, being this difference statistically significant. On the other hand, no statistical differences were observed between groups in the ratio of asymptomatic dogs. Therefore, it has been proved that the likelihood of a dog vaccinated with Neoleish developing clinical signs of the disease was close to 3 times lower than for an unvaccinated dog. Finally, vaccination did not interfere with serological diagnosis and no animal showed any sign of shock nor local or systemic disorder. Neoleish vaccination is well tolerated.

The benefits provided by Neoleish according to this large-scale double-blind randomized field trial can be summarized as high safety profile and proven efficacy. Neoleish didn’t show any adverse effect, including repeated-dose and overdose assays, as well as no effects in pregnant females and the litter sizes. The risk of active infection is reduced by 2-fold and the risk of developing clinical disease decreases three times in Neoleish vaccinated dogs compared to placebo dogs. In addition, vaccinated dogs in which active infection cannot be prevented are expected to present less parasites in bone marrow. In fact, the rate of subpatent infections are approximately double in vaccinated than in control dogs. The risk of parasitemia is also considerably reduced with Neoleish vaccination.

In summary, the contributions of Neoleish, a vaccine with a very high safety profile, to canine leishmaniasis prevention are: i) The odds-ratio for detectable parasites in blood is 3.5 for vaccinated dogs compared to non-vaccinated dogs; if detected, ~86% lower parasitemia levels are expected; ii) A vaccinated dog has a 2-fold lower risk of developing active infection compared to a non-vaccinated dog; in case positive, the parasite load in bone marrow is expected to be ~96% lower; iii) A 3-fold lower risk (~72.7%) of developing clinical disease is expected in a vaccinated compared to a non-vaccinated dog. Neoleish was authorized by the EC based on the EMA opinion to prevent canine leishmaniasis [16]. The simplicity of this DNA vaccine lacking antibiotic resistance genes and the painfulless intranasal delivery method using a nebulizer make this vaccine ideal for the clinical practice to reduce the burden of the disease.

## Supporting information

S1 Table Individual parasite load expressed in Nr. of parasites/mL of bone marrow, in vaccinated dogs. Individual values represent the arithmetic mean of 2 replicas/sample. N.d.: sample not available. † animal withdrawn.
(DOCX)

S2 Table Individual data of parasite loads expressed in Nr. of parasites/mL of blood, in vaccinated and placebo-treated dogs. Individual values represent the arithmetic mean of 2 replicas/sample. † animal withdrawn.
(DOCX)

S3 Table Clinical findings, laboratory blood abnormalities, molecular and serological results in dogs classified with symptomatic infections at the time of the diagnostics and at the end of the study. (DOCX)

S4 Table Proportion (%) of infected, both vaccinated and control dogs, that progressed to leishmaniasis (Symptomatic) or remained without clinical signs (Asymptomatic) throughout the study period. (*p < 0.05, Fisher’s exact test).
(DOCX)

S5 Table Proportion (%) of Inactive infected (subpatent infections) and Non- infected dogs (free of *Leishmania*). Subpatent infections are disclosed in: *(i)* weak PCR positive (below the positive threshold of the assay Ct ≤ 27.0)/ ELISA (Ingezim and/or Civtest) Seronegative, *(ii)* PCR negative/ ELISA Seropositive, and *(iii)* weak PCR+ below the positive threshold (Ct > 27.0)/ ELISA seropositive. Number of subpatent infections were calculated including all the tests performed (*iv*) and only taking into account weak positives to PCR *(v)* (PCR + / ELISA + OR -). Non-infected (*vi*) are dogs negative for all the diagnostic tests (PCR-/ ELISA -). (*p < 0.05, Fisher’s exact test).
(DOCX)

S6 Table Assessment of effect of vaccination on IFAT assay. Days 28 and 208 p.v. IFAT titer and diagnostic interpretation, serial indirect ELISAs results, and Ct values of qPCR are showed. For qPCR the positive threshold is Ct ≤ 27.0.
(DOCX)

S7 Table Pregnant females treated during the study. *Pregnancy periods, 1^st^: from 0 to 22 days of gestation, 2^nd^: 23–44, 3^rd^: 45–65).
(DOCX)

S8 Table Summary of litter sizes by treatment group and number of pregnant females treated during the study, broken down by term of pregnancy. (DOCX)
