# Supplementary material for: Large-scale randomized double-blind field clinical trial for safety and efficacy assessment of the DNA vaccine Neoleish against canine leishmaniasis
Source: PLoS Negl Trop Dis. 2025 Nov 3;19(11):e0012707. doi: 10.1371/journal.pntd.0012707 (PMC12604769; doi:10.1371/journal.pntd.0012707)
Supplement: S1 Table — Individual values represent the arithmetic mean of 2 replicas/sample. N.d.: sample not available. † animal withdrawn. (DOCX) [file pntd.0012707.s001.docx]

**S1 Table. Individual parasite load expressed in Nr. of parasites/mL of bone marrow, in vaccinated dogs.** Individual values represent the arithmetic mean of 2 replicas/sample. N.d.: sample not available. † animal withdrawn.

| **Kennel id.** | **Dog ID** | **Treat.** | **T28 p.v.** | **TT194 p.v.** | **T374 p.v.** | **T574 p.v.** | **T644 p.v.** | **T734 p.v.** |
| --- | --- | --- | --- | --- | --- | --- | --- | --- |
| 1-BA | 941000018249081 | GA | 0.00 | 0.00 | 0.84 | 0.00 | 1.08 | 116.79 |
| 1-BA | 941000018249573 | GA | 0.00 | 0.00 | 0.00 | 0.00 | 1,81 | 0.00 |
| 2-CC | 938000000251466 | GA | 0.00 | 42.38 | 0.00 | 216.184.69 | 0.00 | 0.00 |
| 2-CC | 941000021954803 | GA | 0.00 | 0.00 | 0.00 | 0.00 | 0.00 | 7.36 |
| 2-CC | 981098102897657 | GA | 0.00 | 0.00 | 0.00 | 0.00 | 1.13 | 131.50 |
| 2-CC | 900176000033909 | GA | 0.00 | 0.00 | 0.00 | 0.00 | 0.00 | 5.35 |
| 3-CC | 941000017709459 | GA | 0.00 | 0.00 | 0.00 | 0.00 | 0.11 | 0.00 |
| 3-CC | 941000016790640 | GA | 0.00 | 0.00 | 0.38 | † | † | † |
| 3-CC | 941000017709484 | GA | 0.00 | 0.00 | 0.00 | 119.73 | 105.11 | 6,800.94 |
| 3-CC | 941000019511445 | GA | 0.00 | 0.00 | 0.00 | 2,194.91 | 9.23 | 153,584.20 |
| 3-CC | 981098102885029 | GA | 0.00 | 0.00 | 10.21 | 0.00 | 0.00 | 0.00 |
| 3-CC | 941000021363006 | GA | 0.00 | 0.00 | 312.68 | 0.00 | 0.00 | 0.00 |
| 3-CC | 941000014796713 | GA | 0.00 | 15.54 | 0.00 | 457.61 | 7,462.55 | † |
| 4-CC | 941000014586998 | GA | 0.00 | 0.00 | 0.00 | 3,887.14 | 0.00 | 0,23 |
| 4-CC | 941000016730085 | GA | 9.45 | 75.68 | 105.96 | 1,379.96 | 678.03 | 0.00 |
| 4-CC | 941000016740085 | GA | 0.00 | 0.00 | 0.00 | 0.00 | 0.00 | 8,146.76 |
| 5-CC | 941000017710283 | GA | 0.70 | 1,279.27 | 30.742.98 | 18,348.09 | 0.33 | † |
| 6-BA | 981100002543164 | GA | 0.00 | 0.00 | 0.00 | 0.06 | 0.00 | 0.00 |
| 6-BA | 941000015655247 | GA | 1053.42 | 1,034,298.66 | † | † | † | † |
| 7-BA | 941000012657501 | GA | 0.00 | 0.46 | 16,295.42 | 58,408.75 | 0.00 | 4.26 |
| 8-B | 981098106002666 | GA | 0.00 | 0.00 | 12.97 | 0.00 | † | † |
| 8-B | 8712393079 | GA | 0.00 | 0.00 | 2.50 | 5.40 | 0.00 | 0.00 |
| 8-B | 8712392920 | GA | 0.00 | 0.00 | 335.71 | 127,173.27 | 311,966.77 | 97.35 |
| 8-B | 8712393001 | GA | 0.00 | 0.00 | 6,233.09 | 50,670.80 | 112,609.87 | 2,515.87 |
| 8-B | 8712361567 | GA | 0.00 | 0.00 | 0.00 | 0.00 | 2.19 | 0.00 |
| 8-B | 8712292989 | GA | 0.00 | 0.00 | 6,403.62 | 4,421.86 | 66,112.57 | 8,484.98 |
| 8-B | 8710356136 | GA | 0.00 | 0.00 | 0.00 | 0.00 | 46.02 | 16,365.60 |
| 8-B | 8711137666 | GA | 0.00 | 0.00 | 42.35 | 180.13 | 3.42 | 422.44 |
| 8-B | 8711074408 | GA | 0.00 | 0.00 | 587.24 | 91,192.59 | 252,194.41 | 34,988.62 |
| 8-B | 8711030229 | GA | 0.00 | 0.00 | 0.00 | 392,030.34 | 20,574.79 | 38,395.34 |
| 8-B | 8710377341 | GA | 0.00 | 0.00 | 0.00 | 0.00 | 94.95 | 186.03 |
| 8-B | 8710182815 | GA | 0.00 | 0.00 | 0.00 | 0.00 | 134.74 | 96,155.29 |
| 8-B | 8710190992 | GA | 0.00 | 0.00 | 0.00 | 0.00 | 7,334.03 | 224.49 |
| 8-B | 8710166490 | GA | 0.00 | 0.00 | 0.00 | 232.74 | 7,031.33 | 569.04 |
| 8-B | 8712562870 | GA | 0.00 | 9.43 | 159,715.48 | 107,941.56 | † | † |
| 8-B | 8710339342 | GA | 0.00 | 0.00 | 1,931.88 | 603.53 | 316.76 | 585.15 |
| 8-B | 8710339487 | GA | 0.00 | 0.00 | 129.22 | 124,934.49 | 110,733.09 | 23,219.25 |
| 8-B | 8710339544 | GA | 0.00 | 0.00 | 0.00 | 0.67 | 255.14 | 798.64 |
| 8-B | 8710339457 | GA | 0.00 | 0.00 | 1,171.20 | 109,946.55 | 185,124.62 | 17,468.07 |
| 8-B | 8712563730 | GA | 0.00 | 0.00 | 0.26 | 0.00 | 313.99 | 200.41 |
| 8-B | 8712562986 | GA | 0.00 | 0.00 | 0.00 | 0.00 | 460.24 | 514.49 |
| 8-B | 941000014436400 | GA | 0.00 | 0.00 | 751.48 | 47,983.84 | † | † |
| 8-B | 981098106006956 | GA | 0.00 | 0.00 | 0.00 | 19.23 | 0.00 | 490.09 |
| 8-B | 981098106006261 | GA | 0.00 | 0.00 | 0.00 | 0.00 | 0.00 | 29.69 |
| 8-B | 981098106002956 | GA | 0.00 | 0.00 | 0.00 | 1,322.14 | 0.00 | 0.00 |
| 8-B | 981098106005811 | GA | 0.00 | 0.00 | 0.00 | 0.00 | 1,010.78 | 383,473.61 |
| 8-B | 938000000337973 | GA | 0.00 | 0.00 | 0.00 | 0.00 | 380.59 | 0.00 |
| 8-B | 981098106010578 | GA | 0.00 | 0.00 | 0.00 | 0.00 | 0.00 | 284,831.58 |
| 8-B | 938000000522331 | GA | 0.00 | 0.00 | 0.00 | 0.00 | 0.74 | 12.89 |
| 8-B | 941000014436482 | GA | 0.00 | 0.00 | 0.00 | 0.00 | 0.00 | 78.60 |
| 8-B | 941000015294084 | GA | 0.00 | 0.00 | 0.00 | 42.68 | 4.13 | 3.26 |
| 8-B | 250268711100229 | GA | 0.00 | 0.00 | 0.00 | 0.00 | 0.05 | 0.00 |
| 8-B | 250268712361245 | GA | 0.00 | 0.00 | 0.00 | 0.00 | 1.76 | 6.721.59 |
| 8-B | 250268712360952 | GA | 0.00 | 0.00 | 0.00 | 0.00 | 0.00 | 1.46 |
| 8-B | 250268712361515 | GA | 0.00 | 0.00 | 0.00 | 145,272.77 | 0.00 | † |
| 8-B | 250268712361512 | GA | 0.00 | 0.00 | 0.00 | 0.00 | 1.52 | 0.00 |
| 8-B | 250268712361266 | GA | 0.00 | 0.00 | 0.00 | 0.00 | 0.86 | 0.00 |
| 8-B | 250268712380718 | GA | 0.00 | 0.00 | 0.00 | 24,418.51 | 169,738.35 | 0.00 |
| 8-B | 250268712381908 | GA | 0.00 | 0.00 | 0.00 | 0.00 | 0.27 | 0.00 |

**S1 Table** (continued)

| **Kennel id.** | **Dog ID** | **Treat.** | **T28 p.v.** | **TT194p.v.** | **T374 p.v.** | **T574 p.v.** | **T644 p.v.** | **T734 p.v.** |
| --- | --- | --- | --- | --- | --- | --- | --- | --- |
| 2-CC | 900176000028810 | GB | 0.00 | 0.00 | 0.00 | 12.88 | 2,357,473.00 | 0.00 |
| 2-CC | 938000000447847 | GB | 5.37 | 0.00 | 4.16 | † | † | † |
| 2-CC | 981098104882825 | GB | 0.00 | 0.00 | 0.00 | 0.00 | 0.00 | 27,437.20 |
| 2-CC | 941000017710279 | GB | 0.00 | 0.00 | 0.00 | 0.00 | 28.38 | 60,598.74 |
| 2-CC | 941000016790360 | GB | 0.00 | 0.00 | 0.12 | 0.11 | 84.89 | 0.00 |
| 3-CC | 941000019511095 | GB | 0.00 | 0.00 | 111.29 | 28,987.88 | 195,188.23 | 24,232.99 |
| 3-CC | 941000021362842 | GB | 0.00 | 0.00 | 0.00 | 109.06 | 27,589.41 | 0.00 |
| 4-CC | 941000015763857 | GB | 0.00 | 0.00 | 0.00 | 0.00 | 3,978.67 | 695,263.47 |
| 4-CC | 941000014587002 | GB | 0.00 | 10.72 | 280.53 | † | † | † |
| 5-CC | 941000018252392 | GB | 0.00 | 0.00 | 0.00 | 54.54 | 729.46 | † |
| 5-CC | 941000017709533 | GB | 0.00 | 0.00 | 0.00 | 0.00 | 12,373.40 | 0.00 |
| 7-BA | 941000018589718 | GB | 54.66 | 0.98 | 47.91 | 76.45 | 609.72 | 104.94 |
| 7-BA | 941000017222455 | GB | 0.00 | 0.00 | 1,549.91 | 42,429.55 | 1,903,522.88 | 864,075.47 |
| 7-BA | 941000018594616 | GB | 0.00 | 0.00 | 0.00 | 0.00 | 3.81 | 0.00 |
| 7-BA | 941000018594325 | GB | 0.00 | 0.00 | 0.12 | 0.00 | 0.12 | 602,867.31 |
| 8-B | 8712392912 | GB | 0.00 | 0.00 | 8.97 | 0.00 | 0.00 | 0.00 |
| 8-B | 8712392913 | GB | 0.00 | 0.00 | 0.00 | 0.00 | 3.58 | 0.00 |
| 8-B | 8712392953 | GB | 0.00 | 0.00 | 25.84 | 0.00 | 277,083.88 | 115,305.71 |
| 8-B | 8712428592 | GB | 0.00 | 0.00 | 0.00 | 0.00 | 0.00 | 0.00 |
| 8-B | 8712392908 | GB | 0.00 | 7.89 | 1,619,105.81 | 6,149.48 | 71,048.09 | 54,201.41 |
| 8-B | 8712307504 | GB | 0.00 | 0.00 | 16.06 | 2,144.18 | 224,083.77 | 152,912.75 |
| 8-B | 8712292946 | GB | 0.00 | 0.00 | 89.01 | 0.00 | 503.00 | 17,974.37 |
| 8-B | 8712361376 | GB | 0.00 | 0.00 | 0.00 | 0.00 | 0.88 | 0.00 |
| 8-B | 8712291746 | GB | 0.00 | 0.00 | 2,720.90 | 51,740.39 | 804.263,09 | 177,030.80 |
| 8-B | 8712293319 | GB | 0.00 | 0.00 | 0.00 | 0.00 | 2.45 | 0.00 |
| 8-B | 8712284903 | GB | 0.00 | 0.00 | 0.00 | 1,379.96 | 42,993.63 | 132.27 |
| 8-B | 8710339297 | GB | 0.00 | 0.00 | 234,920.84 | 31,759.12 | 154,334.91 | 55,823.20 |
| 8-B | 9500573076 | GB | 0.00 | 0.00 | 0.00 | 0.00 | 444.77 | 113,840.55 |
| 8-B | 8601026760 | GB | 0.00 | 0.00 | 0.00 | 23.09 | 154.88 | 222,856.30 |
| 8-B | 8710219028 | GB | 0.00 | 0.00 | 0.00 | 3,301.62 | 72,953.99 | 275,632.97 |
| 8-B | 8710182613 | GB | 0.00 | 0.00 | 63.60 | 11.24 | 797.34 | 271,447.27 |
| 8-B | 8710339600 | GB | 0.00 | 0.00 | 0.00 | 0.00 | 0.00 | 0.00 |
| 8-B | 8710339489 | GB | 0.00 | 0.00 | 0.00 | 130,664.13 | 0.00 | 225,688.19 |
| 8-B | 8710339490 | GB | 0.00 | 0.00 | 219.43 | 207,135.30 | 51,736.74 | 18,213.00 |
| 8-B | 8712562862 | GB | 0.00 | 0.00 | 0.00 | 958.41 | 47,886.00 | 276,531.94 |
| 8-B | 8712562978 | GB | 0.00 | 0.00 | 0.00 | 1,148.53 | 203,661.60 | 33,113.45 |
| 8-B | 8710339456 | GB | 0.00 | 0.00 | 42,219.57 | † | † | † |
| 8-B | 8712563716 | GB | 5.52 | 0.00 | 8,293.01 | 191,908.20 | 114,716.44 | 24,521.46 |
| 8-B | 941000015294258 | GB | 0.00 | 0.00 | 491.16 | 42,692.57 | 315,045.34 | 196,347.78 |
| 8-B | 981098106004812 | GB | 0.00 | 0.00 | 0.00 | 4.74 | 0.00 | 606.85 |
| 8-B | 981098106000607 | GB | 0.00 | 0.00 | 0.00 | 0.00 | n.d. | 11.56 |
| 8-B | 981098106010086 | GB | 0.00 | 0.00 | 6.60 | 759,052.16 | 430,611.13 | 520,187.70 |
| 8-B | 941000015293897 | GB | 0.00 | 0.00 | 0.00 | 0.00 | 0.00 | 9.33 |
| 8-B | 938000000371011 | GB | 0.00 | 0.00 | 52.06 | 389.50 | 3,807.90 | 16,942.57 |
| 8-B | 981098106000936 | GB | 0.00 | 0.00 | 0.00 | 453.28 | n.d. | 622,829.81 |
| 8-B | 981098106000944 | GB | 0.00 | 0.00 | 0.00 | 162.29 | 291,826.56 | 1,072.88 |
| 8-B | 981098106008105 | GB | 0.00 | 0.00 | 184.77 | 71,963.07 | 291,826.56 | 922,232.28 |
| 8-B | 250268720036515 | GB | 0.00 | 0.00 | 0.00 | 0.00 | 291,826.56 | 22,76 |
| 8-B | 981098106010638 | GB | 0.00 | 0.00 | 0.00 | 137.64 | † | † |
| 8-B | 941000016440434 | GB | 0.00 | 0.00 | 0.00 | 0.00 | 4,825,071.75 | 192,595.43 |
| 8-B | 250268711100207 | GB | 0.00 | 0.00 | 0.00 | 0.00 | 0.67 | 0.00 |
| 8-B | 941000016440441 | GB | 0.00 | 0.00 | 0.00 | 0.00 | 481.55 | 0.00 |
