# Supplementary material for: Large-scale randomized double-blind field clinical trial for safety and efficacy assessment of the DNA vaccine Neoleish against canine leishmaniasis
Source: PLoS Negl Trop Dis. 2025 Nov 3;19(11):e0012707. doi: 10.1371/journal.pntd.0012707 (PMC12604769; doi:10.1371/journal.pntd.0012707)
Supplement: S2 Table — Individual values represent the arithmetic mean of 2 replicas/sample. † animal withdrawn. (DOCX) [file pntd.0012707.s002.docx]

**S2 Table. Individual data of parasite loads expressed in Nr. of parasites/mL of blood, in vaccinated and placebo-treated dogs.** Individual values represent the arithmetic mean of 2 replicas/sample. † animal withdrawn.

| **Kennel id.** | **Dog ID** | **Treat** | **T28 p.v.** | **TT194 p.v.** | **T374 p.v.** | **T574 p.v.** | **T644 p.v.** | **T734 p.v.** |
| --- | --- | --- | --- | --- | --- | --- | --- | --- |
| 1-BA | 941000018249081 | GA | 0.00 | 0.00 | 0.00 | 0.00 | 0.00 | 0.00 |
| 1-BA | 941000018249573 | GA | 0.00 | 0.00 | 0.00 | 0.00 | 0.00 | 0.00 |
| 2-CC | 938000000251466 | GA | 0.00 | 0.00 | 0.00 | 99.58 | 0.00 | 0.00 |
| 2-CC | 941000021954803 | GA | 0.00 | 0.00 | 0.00 | 0.00 | 0.00 | 0.00 |
| 2-CC | 981098102897657 | GA | 0.00 | 0.00 | 0.00 | 0.00 | 0.00 | 0.00 |
| 2-CC | 900176000033909 | GA | 0.00 | 0.00 | 0.00 | 0.00 | 0.00 | 0.00 |
| 3-CC | 941000017709459 | GA | 0.00 | 0.00 | 0.00 | 0.00 | 0.00 | 0.00 |
| 3-CC | 941000016790640 | GA | 0.00 | 0.00 | 0.00 | † | † | † |
| 3-CC | 941000017709484 | GA | 0.00 | 0.00 | 0.00 | 0.00 | 0.00 | 0.00 |
| 3-CC | 941000019511445 | GA | 0.00 | 0.00 | 0.00 | 0.21 | 0.00 | 0.70 |
| 3-CC | 981098102885029 | GA | 0.00 | 0.00 | 0.00 | 0.00 | 0.00 | 0.00 |
| 3-CC | 941000021363006 | GA | 0.00 | 0.00 | 0.00 | 0.00 | 0.00 | 0.00 |
| 3-CC | 941000014796713 | GA | 0.00 | 0.00 | 0.00 | 0.00 | 0.38 | † |
| 4-CC | 941000014586998 | GA | 0.00 | 0.00 | 0.00 | 0.00 | 0.00 | 0.00 |
| 4-CC | 941000016730085 | GA | 0.00 | 0.00 | 0.28 | 0.00 | 0.00 | 0.00 |
| 4-CC | 941000016740085 | GA | 0.00 | 0.00 | 0.00 | 0.00 | 0.00 | 0.00 |
| 5-CC | 941000017710283 | GA | 0.00 | 0.00 | 245.10 | 3.35 | 0.00 | † |
| 6-BA | 981100002543164 | GA | 0.00 | 0.00 | 0.00 | 0.00 | 0.00 | 0.00 |
| 6-BA | 941000015655247 | GA | 0.00 | 28.92 | † | † | † | † |
| 7-BA | 941000012657501 | GA | 0.00 | 0.00 | 0.00 | 0.00 | 0.00 | 0.00 |
| 8-B | 981098106002666 | GA | 0.00 | 0.00 | 0.00 | 0.00 | † | † |
| 8-B | 8712393079 | GA | 0.00 | 0.00 | 0.00 | 0.00 | 0.00 | 0.00 |
| 8-B | 8712392920 | GA | 0.00 | 0.00 | 46.69 | 30.75 | 190.84 | 0.10 |
| 8-B | 8712393001 | GA | 0.00 | 0.00 | 52.26 | 20.86 | 2.68 | 0.00 |
| 8-B | 8712361567 | GA | 0.00 | 0.00 | 0.00 | 0.00 | 0.00 | 0.00 |
| 8-B | 8712292989 | GA | 0.00 | 0.00 | 0.12 | 0.00 | 106.92 | 0.00 |
| 8-B | 8710356136 | GA | 0.00 | 0.00 | 0.00 | 0.00 | 0.00 | 0.00 |
| 8-B | 8711137666 | GA | 0.00 | 0.00 | 0.00 | 0.00 | 0.00 | 0.00 |
| 8-B | 8711074408 | GA | 0.00 | 0.00 | 0.00 | 0.00 | 76.83 | 12.95 |
| 8-B | 8711030229 | GA | 0.00 | 0.00 | 0.00 | 30.51 | 43.97 | 35.01 |
| 8-B | 8710377341 | GA | 0.00 | 0.00 | 0.00 | 0.00 | 0.00 | 0.00 |
| 8-B | 8710182815 | GA | 0.00 | 0.00 | 0.00 | 0.00 | 0.00 | 126.19 |
| 8-B | 8710190992 | GA | 0.00 | 0.00 | 0.00 | 0.00 | 1.34 | 14.70 |
| 8-B | 8710166490 | GA | 0.00 | 0.00 | 0.00 | 0.00 | 1.06 | 0.00 |
| 8-B | 8712562870 | GA | 0.00 | 0.00 | 59.18 | 102.12 | † | † |
| 8-B | 8710339342 | GA | 0.00 | 0.00 | 0.00 | 0.00 | 0.00 | 6.69 |
| 8-B | 8710339487 | GA | 0.00 | 0.00 | 0.00 | 8.71 | 52.76 | 81.82 |
| 8-B | 8710339544 | GA | 0.00 | 0.00 | 0.00 | 0.00 | 0.00 | 0.00 |
| 8-B | 8710339457 | GA | 0.00 | 0.00 | 0.00 | 12.24 | 6.02 | 6.21 |
| 8-B | 8712563730 | GA | 0.00 | 0.00 | 0.00 | 0.00 | 0.00 | 0.00 |
| 8-B | 8712562986 | GA | 0.00 | 0.00 | 0.00 | 0.00 | 0.00 | 0.00 |
| 8-B | 941000014436400 | GA | 0.00 | 0.00 | 0.00 | 8.03 | † | † |
| 8-B | 981098106006956 | GA | 0.00 | 0.00 | 0.00 | 0.00 | 0.00 | 0.00 |
| 8-B | 981098106006261 | GA | 0.00 | 0.00 | 0.00 | 0.00 | 0.00 | 0.00 |
| 8-B | 981098106002956 | GA | 0.00 | 0.00 | 0.00 | 0.00 | 0.00 | 0.00 |
| 8-B | 981098106005811 | GA | 0.00 | 0.00 | 0.00 | 0.00 | 0.00 | 111.56 |
| 8-B | 938000000337973 | GA | 0.00 | 0.00 | 0.00 | 0.00 | 0.00 | 0.00 |
| 8-B | 981098106010578 | GA | 0.00 | 0.00 | 0.00 | 0.00 | 0.00 | 511.81 |
| 8-B | 938000000522331 | GA | 0.00 | 0.00 | 0.00 | 0.00 | 0.00 | 0.00 |
| 8-B | 941000014436482 | GA | 0.00 | 0.00 | 0.00 | 0.00 | 0.00 | 0.00 |
| 8-B | 941000015294084 | GA | 0.00 | 0.00 | 0.00 | 0.00 | 0.00 | 0.00 |
| 8-B | 250268711100229 | GA | 0.00 | 0.00 | 0.00 | 0.00 | 0.00 | 0.00 |
| 8-B | 250268712361245 | GA | 0.00 | 0.00 | 0.00 | 0.00 | 0.00 | 0.00 |
| 8-B | 250268712360952 | GA | 0.00 | 0.00 | 0.00 | 0.00 | 0.00 | 0.00 |
| 8-B | 250268712361515 | GA | 0.00 | 0.00 | 0.47 | 10.42 | 0.00 | † |
| 8-B | 250268712361512 | GA | 0.00 | 0.00 | 0.00 | 0.00 | 0.00 | 0.00 |
| 8-B | 250268712361266 | GA | 0.00 | 0.00 | 0.00 | 0.00 | 0.00 | 0.00 |
| 8-B | 250268712380718 | GA | 0.00 | 0.00 | 0.00 | 0.00 | 0.08 | 0.00 |
| 8-B | 250268712381908 | GA | 0.00 | 0.00 | 0.00 | 0.00 | 0.00 | 0.00 |

**S2 Table** (continued)

| **Kennel id.** | **Dog ID** | **Treat.** | **T28 p.v.** | **TT194 p.v.** | **T374 p.v.** | **T574 p.v.** | **T644 p.v.** | **T734 p.v.** |
| --- | --- | --- | --- | --- | --- | --- | --- | --- |
| 2-CC | 900176000028810 | GB | 0.00 | 0.00 | 0.00 | 0.00 | 822.83 | 0.00 |
| 2-CC | 938000000447847 | GB | 0.00 | 0.00 | 0.00 | † | † | † |
| 2-CC | 981098104882825 | GB | 0.00 | 0.00 | 0.00 | 0.00 | 0.00 | 93.18 |
| 2-CC | 941000017710279 | GB | 0.00 | 0.00 | 0.00 | 0.00 | 0.00 | 47.55 |
| 2-CC | 941000016790360 | GB | 0.00 | 0.00 | 0.00 | 0.00 | 0.00 | 0.00 |
| 3-CC | 941000019511095 | GB | 0.00 | 0.00 | 0.00 | 0.00 | 1.47 | 0.10 |
| 3-CC | 941000021362842 | GB | 0.00 | 0.00 | 0.00 | 0.00 | 1.23 | 0.00 |
| 4-CC | 941000015763857 | GB | 0.00 | 0.00 | 0.00 | 0.00 | 0.00 | 89.03 |
| 4-CC | 941000014587002 | GB | 0.00 | 0.00 | 0.00 | † | † | † |
| 5-CC | 941000018252392 | GB | 0.00 | 0.00 | 0.00 | 0.00 | 0.00 | † |
| 5-CC | 941000017709533 | GB | 0.00 | 0.00 | 0.00 | 0.00 | 0.00 | 0.00 |
| 7-BA | 941000018589718 | GB | 0.00 | 0.00 | 0.00 | 0.00 | 0.00 | 0.00 |
| 7-BA | 941000017222455 | GB | 0.00 | 0.00 | 0.00 | 0.00 | 0.00 | 6.28 |
| 7-BA | 941000018594616 | GB | 0.00 | 0.00 | 0.00 | 0.00 | 0.00 | 0.00 |
| 7-BA | 941000018594325 | GB | 0.00 | 0.00 | 0.00 | 0.00 | 0.00 | 0.00 |
| 8-B | 8712392912 | GB | 0.00 | 0.00 | 0.00 | 0.00 | 0.00 | 0.00 |
| 8-B | 8712392913 | GB | 0.00 | 0.00 | 0.00 | 0.00 | 0.00 | 0.00 |
| 8-B | 8712392953 | GB | 0.00 | 0.00 | 0.00 | 0.00 | 136.87 | 263.23 |
| 8-B | 8712428592 | GB | 0.00 | 0.00 | 0.00 | 0.00 | 0.00 | 0.00 |
| 8-B | 8712392908 | GB | 0.00 | 0.00 | 159.53 | 4.61 | 303.88 | 5.15 |
| 8-B | 8712307504 | GB | 0.00 | 0.00 | 0.00 | 0.00 | 266.61 | 478.95 |
| 8-B | 8712292946 | GB | 0.00 | 0.00 | 0.00 | 0.00 | 0.00 | 101.96 |
| 8-B | 8712361376 | GB | 0.00 | 0.00 | 0.00 | 0.00 | 0.00 | 0.00 |
| 8-B | 8712291746 | GB | 0.00 | 0.00 | 0.00 | 191.82 | 275.70 | 557.17 |
| 8-B | 8712293319 | GB | 0.00 | 0.00 | 0.00 | 0.00 | 0.00 | 0.00 |
| 8-B | 8712284903 | GB | 0.00 | 0.00 | 0.00 | 0.00 | 4.97 | 0.00 |
| 8-B | 8710339297 | GB | 0.00 | 0.00 | 87.97 | 0.00 | 22.28 | 23.20 |
| 8-B | 9500573076 | GB | 0.00 | 0.00 | 0.00 | 0.00 | 0.00 | 53.27 |
| 8-B | 8601026760 | GB | 0.00 | 0.00 | 0.00 | 3.45 | 0.00 | 293.62 |
| 8-B | 8710219028 | GB | 0.00 | 0.00 | 0.00 | 0.00 | 40.68 | 201.45 |
| 8-B | 8710182613 | GB | 0.00 | 0.00 | 0.00 | 0.00 | 0.00 | 201.44 |
| 8-B | 8710339600 | GB | 0.00 | 0.00 | 0.00 | 0.00 | 0.00 | 0.00 |
| 8-B | 8710339489 | GB | 0.00 | 0.00 | 0.00 | 0.00 | 0.00 | n.d. |
| 8-B | 8710339490 | GB | 0.00 | 0.00 | 0.04 | 7.16 | 183.37 | 2.79 |
| 8-B | 8712562862 | GB | 0.00 | 0.00 | 0.00 | 0.00 | 0.00 | 43.96 |
| 8-B | 8712562978 | GB | 0.00 | 0.00 | 0.00 | 0.15 | 542.14 | 5.86 |
| 8-B | 8710339456 | GB | 0.00 | 0.00 | 0.00 | † | † | † |
| 8-B | 8712563716 | GB | 0.00 | 0.00 | 1.92 | 2.83 | 223.99 | 70.63 |
| 8-B | 941000015294258 | GB | 0.00 | 0.00 | 0.00 | 1.50 | 558.50 | 337.24 |
| 8-B | 981098106004812 | GB | 0.00 | 0.00 | 0.00 | 0.00 | 0.00 | 6.64 |
| 8-B | 981098106000607 | GB | 0.00 | 0.00 | 0.00 | 0.00 | n.d. | 15.51 |
| 8-B | 981098106010086 | GB | 0.00 | 0.00 | 0.00 | 107.87 | 23.85 | 39,690.26 |
| 8-B | 941000015293897 | GB | 0.00 | 0.00 | 0.00 | 0.00 | 0.00 | n.d. |
| 8-B | 938000000371011 | GB | 0.00 | 0.00 | 0.00 | 0.00 | 0.00 | 32.61 |
| 8-B | 981098106000936 | GB | 0.00 | 0.00 | 0.00 | 0.00 | 13.07 | 330.21 |
| 8-B | 981098106000944 | GB | 0.00 | 0.00 | 0.00 | 0.00 | 0.00 | 0.00 |
| 8-B | 981098106008105 | GB | 0.00 | 0.00 | 0.00 | 18.62 | 5,461.92 | 425.07 |
| 8-B | 250268720036515 | GB | 0.00 | 0.00 | 0.00 | 0.00 | 0.00 | 0.37 |
| 8-B | 981098106010638 | GB | 0.00 | 0.00 | 0.00 | 0.00 | † | † |
| 8-B | 941000016440434 | GB | 0.00 | 0.00 | 0.00 | 0.00 | 942.71 | 90.02 |
| 8-B | 250268711100207 | GB | 0.00 | 0.00 | 0.00 | 0.00 | 0.00 | 0.00 |
| 8-B | 941000016440441 | GB | 0.00 | 0.00 | 0.00 | 0.00 | 0.00 | 0.00 |
