# Supplementary material for: Large-scale randomized double-blind field clinical trial for safety and efficacy assessment of the DNA vaccine Neoleish against canine leishmaniasis
Source: PLoS Negl Trop Dis. 2025 Nov 3;19(11):e0012707. doi: 10.1371/journal.pntd.0012707 (PMC12604769; doi:10.1371/journal.pntd.0012707)
Supplement: S3 Table — (DOCX) [file pntd.0012707.s003.docx]

**S3 Table. Clinical findings, laboratory blood abnormalities, molecular and serological results in dogs classified with symptomatic infections at the time of the diagnostics and at the end of the study.**

| **Kennel id** | **Dog Id.** | **Treat. group** | **Last d.c.s. *(Nr. Of clinical signs + Nr. Of para-clinical***  ***signs)*** | **1^st^ PCR / Parasite load detected in b.m. (N/mL)** | **Last Clinical sign (score grade), para-clinic deviation observed (±physiological range*), *[ ]* para-clinical score**, **parasite burden** (bone marrow and blood).** | **Last serology available (O.D. Ingezim ELISA/ Rz titer CivTest ELISA / IFAT equivalence with CivTest** |
| --- | --- | --- | --- | --- | --- | --- |
| 8-B | 8712562870 | Vacc | 12  *(3+3)* | T194, Load: 9.43 | Withdrawn (Date:22/10/2018), Low body condition (1), anaemic mucous membranes (1), muscular-cephalic atrophy (1), ALB=1,8 (2,6 - 4) g/dl [*3*], BUN=59,0 (9,2 - 29,2)* mg/dl *[3],*  LDH=590 (24-219) IU/L *[3].* At T544 (last sampling available): qPCR B.m. Load**=107941.56 N/mL, qPCR Blood load** =102.12 N/mL | O.D. ELISA=3,118, Rz=9,390, IFAT>1/640 |
| 8-B | 8710339487 | Vacc | 11  *(4+2)* | T374. Load:129.22 | T734: Low body condition (1), anaemic mucous membranes (1), muscular-cephalic atrophy (1), alopecia (4), ALB=1,9 (2,6-4) g/dl *[3],* CREA=0,3 (0,4-1,4) mg/dl *[1],* At T734: B.m. Load: 2,515,87,  Load blood=0 | DO. ELISA 3,348, Rz=4,07, IFAT>1/640 |
| 8-B | 8712393001 | Vacc | 12  *(5+2)* | T734, Load:6,23 | T734: Low body condition (1), anaemic mucous membranes (1), muscular-cephalic atrophy (1), alopecia(2), Exfoliative dermatitis (4), ALB=2,4 (2,6-4) g/dl *[1],* T-PRO=8,3 (5,5-7,5) g/dl *[2].* At T734: B.m. Load=2,515.85, Blood load =0 | O.D. ELISA=0,721, Rz=5,155, IFAT>1/640 |
| 8-B | 8711030229 | Vacc | 15  *(6+4)* | T554, Load:392030.34. | T734: Low body condition (1), anaemic mucous membranes (1), muscular-cephalic atrophy (1), lymphadenopathy (2), alopecia (2), weight loss (1). ALB=1.3 (2.6-4) g/dl *[3]*, ALP=92 (13-83) IU/L  *[2]*, CREA=0.3 (0,4-1,4) mg/dl *[1]*, T-PRO=4.4 (5.5-7.5) g/dl *[3],* B.m. Load=38395.34, Blood load  =35.01 | O.D. ELISA=0.625, Rz=5.15, IFAT>1/640 |
| 8-B | 941000014436400 | Vacc | 15  *(5+3)* | T374, Load: 751.48 | Withdrawn (Date: 21/12/2018), Low body condition (1), anaemic mucous membranes (1), muscular-cephalic atrophy (1), alopecia (2), Exfoliative dermatitis (4). ALB=2.3 (2.6 - 4) g/dl *[2]*,  BUN=36.9 (9.2 - 29.2) mg/dl *[3]*, T-PRO=7.9 (5.5 - 7.5) g/dl *[1]*. At T554 (last sampling available):  B.m. Load=47,983, Blood load =8.03. | O.D. ELISA=1.901, Rz=4.540, IFAT >1/640. |
| 2-CC | 941000017710279 | Contr | 11  *(3+3)* | T644, Load:28.38 | T734: Muscular-cephalic atrophy (1), lymphadenopathy (1), alopecia (2), ALB=1.8 (2.6-4) g/dl *[3]*,  LDH=580 (24-219) IU/L *[3]*, Total-P=7.62 (5.5 - 7.5) g/dl *[1]*. At T734: B.m. Load=60598,74, B.m.  Blood load =47.55. | ELISA Negative |
| 7-BA | 941000018589718 | Contr | 9  *(3+3)* | T28, Load: 54.66 | T734: Muscular-cephalic atrophy (1), lymphadenopathy (1), alopecia (2), ALB=4.4 (2.6-4) g/dl *[1],*  T-PROT=8.12 (5.5 - 7.5) g/dl *[1]*, UREA=33 (8.8-26 mg/dl) *[3]*. At T734: B.m. Load=104.94, Blood  load =0 | ELISA Negative |
| 8-B | 981098106008105 | Contr | 13  *(3+3)* | T374, Load: 184.77 | T734: Low body condition (1), muscular-cephalic atrophy (1), alopecia (2), ALB=1,5 (2,6-4) g/dl *[3],*  T-PRO=1,2 (5,5 - 7,5) g/dl *[3]*, CREAT=0,2 (0,4-1,4) mg/dl *[3].* At T734: B.m. Load=922,232.28,  Blood load =425.07. | O.D. ELISA: 2.797, Rz:8.242, IFAT=>1/640 |
| 8-B | 981098106010086 | Contr | 12  *(2+4)* | T374, Load:6.6 | T734: Low body condition (1), muscular-cephalic atrophy (1), ALB=1,3 (2,6-4) g/dl *[3],* CREAT=0,3  (0,4-1,4) mg/dl *[3]*, GPT(ALT)=112 (17-78) IU/L *[3],*, LDH=259 (24-219) IU/L *[1]*. At T734: B.m.  Load=520,187.70, Blood load =39.690, | O.D. ELISA=2.918. Rz=8.830, IFAT>1/640 |
| 8-B | 8710339489 | Contr | 11  *(3+3)* | T544, Load: 130664,13 | T734: Low body condition (1), Uveitis (2), ulcers (1), ALB=2.4 (2.6-4) g/dl *[1]*, T-PRO:4.6 (5.5-7.5) g/dl *[3],* LDH=170 (24-219) IU/L *[3].* At T734: B.m. Load=225,688.97, Blood load =n.d. | O.D. ELISA=0.531, Rz=0.567, IFAT=1/40. |
| 8-B | 8710339456 | Contr | 12  *(3+3)* | T374, Load: 42219,57 | Withdrawn (Date:02/07/2018), anaemic mucous membranes (1), onychogryphosis (1), muscular- cephalic atrophy (1), ALB=0.9 (2.6 - 4) g/dl *[3]*, BUN=7 (8.8 - 26) mg/dl *[3]*, T-PRO=2.5 (5.5 - 7.5)  g/dl *[3]*. At T374 (last sampling available): B.m. Load=42219/57, Blood load =0. | O.D. ELISA=1.902, Rz=4.91, IFAT=>1/640 |

**S3 Table** (continues).

| **Kennel id** | **Dog Id.** | **Treat. group** | **Last d.c.s. *(Nr. Of clinical signs***  ***+ Nr. Of para- clinical signs)*** | **1^st^ PCR / Parasite load detected in**  **b.m. (N/mL)** | **Last Clinical sign (score grade), para-clinic deviation observed (±physiological range*), *[ ]* para-clinical score**, **parasite burden** (bone marrow and blood).** | **Last serology available (O.D. Ingezim ELISA/ Rz titer CivTest ELISA / IFAT**  **equivalence with CivTest** |
| --- | --- | --- | --- | --- | --- | --- |
| 5-CC | 941000018252392 | Contr | 13  *(3+3)* | T554, Load:54.54. | Withdrawn (Date: 01/02/2019), muscular-cephalic atrophy (1), lymphadenopathy (2), ulcers (2), ALB=1,6 (2,6-4) g/dl *[3]*, GOT=57 (8,9 - 48,5) IU/L *[3]*, UREA=29 (8,8-26)  mg/dl *[2]*. At T644 (last sampling available): B.m. Load: 12373, 40. B.m. Blood load =0. | ELISA Negative |
| 7-BA | 941000017222455 | Contr | 13  *(2+3)* | T374, Load:1549.91 | T734: Lymphadenopathy (2), alopecia (2), LDH=306 (24 - 219) IU/L *[3]*, T-PRO =10.62  (5.5 - 7,5) g/dl *[3]*, UREA=38 (8.8-26) mg/dl *[3]*. At T734: B.m. Load=864075.47, Blood  load =6.28. | O.D. ELISA=3.329. Rz=6.620, IFAT=>1/640 |
| 8-B | 8710339297 | Contr | 14  *(4+3)* | T374, Load:234920.84 | T734: Low body condition (1), anaemic mucous membranes (1), muscular-cephalic atrophy (1), uveitis (2), ALB=<1,0 (2,6-4) g/dl *[3]*, T-PRO=3,4 (5,5-7,5) g/dl *[3]*, LDH=259  (24-219) IU/L *[3]*. At T734: B.m. Load=55.823. 20, Blood load= 23.20 | O.D. ELISA=0.499, Rz=4.804, IFAT=>1/640 |
| 2-CC | 938000000447847 | Contr | 13  *(5+3)* | T28, Load:5.37 | Withdrawn (Date: 10/06/18). Low body condition (1), lymphadenopathy (2), Alopecia (2), Ulcers (1), Exfoliative dermatitis (2). ALB=2.1 (2.6-4) g/dl *[1]*, T-PRO=10.22 (5.5-7.5) g/dl *[3]*, UREA=28 (8.8-26) mg/dl *[1]*. At T374 (last sampling available): B.m. Load=4.16, Blood load =0. | ELISA Negative |
| 8-B | 8710219028 | Contr | 10  *(3+4)* | T544, Load:3301.62 | T734: Low body condition (1), muscular-cephalic atrophy (1), conjunctivitis (2), ALB=2.4 (2.6-4) g/dl *[1]*, ALP=105 (13-83) IU/L *[3]*, BUN=8.6 (9.2-29.2) mg/dl *[1]*, T-PRO=7.8 (5.5-  7.5) g/dl *[1]*. At T734: B.m. Load=275,632.97, B.m. Blood load=201.45. | O.D. ELISA=0.453, Rz=5.103, IFAT>1/640 |
| 8-B | 938000000371011 | Contr | 8  *(3+5)* | T374, Load: 52.06 | T734: Low body condition (1), anaemic mucous membranes (1), muscular-cephalic atrophy (1), ALB=1.3 (2.6-4) g/dl *[3]*, BUN=6.5 (9.2-29.2) mg/dl *[3]*, CREA=0.3 (0.4-1.4)  mg/dl *[3]*, LDH=482 (24-219) IU/L *[3]*, T-PRO=3.9 (5.5-7.5) g/dl *[3]*. At T734: B.m.  Load=16,942.57, Blood load =32.61. | O.D. ELISA: 0.605, Rz=1.569, IFAT=1/160 to 1/320 |
| 8-B | 8712392908 | Contr | 18  *(5+3)* | T198, Load:7.89. | T734: Low body condition (1), anaemic mucous membranes (1), muscular-cephalic atrophy (1), lymphadenopathy (1), alopecia (2), ALB=1.7 (2.6-4) g/dl *[3]*, ALP=109 (13-  83) IU/L *[3]*, BUN=64 (9.2-29.2) mg/dl *[3]*. At T347: B.m. Load=54,201.41, Blood load  =5.15. | O.D. ELISA=0.687, Rz=5.05, IFAT=>1/640 |
| 8-B | 941000016440434 | Contr | 14  *(4+4)* | T644, Load: 5825.07 | T734: Muscular-cephalic atrophy, lymphadenopathy (1), alopecia (2), exfoliative dermatitis (1), ALB=1.8 (2.6-4) g/dl *[3]*, BUN=6.7 (9.2-29.2) mg/dl *[3]*, CREA=0.3 (0.4-1.4)  mg/dl *[3]*, GPT(ALT)=114 (17-78) IU/L *[3]*. At T734: B.m. Load=192,595.43, Blood load: 90.02. | O.D. ELISA: 2.607, Rz=6.837, IFAT=>1/640 |
