# Supplementary material for: Large-scale randomized double-blind field clinical trial for safety and efficacy assessment of the DNA vaccine Neoleish against canine leishmaniasis
Source: PLoS Negl Trop Dis. 2025 Nov 3;19(11):e0012707. doi: 10.1371/journal.pntd.0012707 (PMC12604769; doi:10.1371/journal.pntd.0012707)
Supplement: S4 Table — (*p < 0.05, Fisher’s exact test). (DOCX) [file pntd.0012707.s004.docx]

**S4 Table. Proportion (%) of infected, both vaccinated and control dogs, that progressed to leishmaniasis (Symptomatic) or remained without clinical signs (Asymptomatic) throughout the study period.** (*p<0.05, Fisher’s exact test).

| **T p.v.** | | **Vaccinated (GA)** | | | **Controls (GB)** | | |  |
| --- | --- | --- | --- | --- | --- | --- | --- | --- |
|  |  | **Nr.** | **N total** | **%** | **Nr.** | **N total** | **%** | **p**  **(OR, 95%CI)** |
|  | **T28** | 0 | 178 | 0.00 | 0 | 179 | 0.00 | -- |
|  | **T194** | 0 | 171 | 0.00 | 0 | 175 | 0.00 | -- |
|  | **T374** | 0 | 162 | 0.00 | 1 | 162 | 0.62 | 0.51 |
| **Symptomatic** | **T554** | 5 | 153 | 3.27 | 5 | 151 | 3.31 | 0.61 |
|  | **T644** | 4 | 145 | 2.76 | 7 | 148 | 4.73 | 0.29 |
|  | **T734** | 3 | 141 | 2.13 | 11 | 141 | 7.80 | 0.0327* |
|  |  |  |  |  |  |  |  | (OR: 3.89, 1.06-14.26) |
|  | **LOCF** | 5 | 181 | 2.76 | 14 | 180 | 7.78 | 0.035* |
|  |  |  |  |  |  |  |  | (OR: 2.96, 1.04-8.42) |
| **Asymptomatic** | **T28** | 1 | 178 | 0.56 | 0 | 179 | 0.00 | 0.50 |
|  | **T194** | 1 | 171 | 0.58 | 0 | 175 | 0.00 | 0.49 |
|  | **T374** | 11 | 162 | 6.79 | 8 | 162 | 4.94 | 0.50 |
|  | **T554** | 16 | 153 | 10.46 | 13 | 151 | 8.61 | 0.38 |
|  | **T644** | 13 | 145 | 8.97 | 22 | 148 | 14.86 | 0.11 |
|  | **T734** | 11 | 141 | 7.80 | 17 | 141 | 12.06 | 0.18 |
|  | **LOCF** | 13 | 181 | 7.18 | 18 | 180 | 10.00 | 0.24 |
