# Supplementary material for: Large-scale randomized double-blind field clinical trial for safety and efficacy assessment of the DNA vaccine Neoleish against canine leishmaniasis
Source: PLoS Negl Trop Dis. 2025 Nov 3;19(11):e0012707. doi: 10.1371/journal.pntd.0012707 (PMC12604769; doi:10.1371/journal.pntd.0012707)
Supplement: S5 Table — Subpatent infections are disclosed in: (i) weak PCR positive (below the positive threshold of the assay Ct ≤ 27.0)/ ELISA (Ingezim and/or Civtest) Seronegative, (ii) PCR negative/ ELISA Seropositive, and (iii) weak PCR+ below the positive threshold (Ct > 27.0)/ ELISA seropositive. Number of subpatent infections were calculated including all the tests performed (iv) and only taking into account weak positives to PCR (v) (PCR + / ELISA + OR -). Non-infected (vi) are dogs negative for all the diagnostic tests (PCR-/ ELISA -). (*p < 0.05, Fisher’s exact test). (DOCX) [file pntd.0012707.s005.docx]

### S5 Table. Proportion (%) of Inactive infected (subpatent infections) and Non- infected dogs (free of *Leishmania*). Subpatent infections are disclosed in: *(i)* weak PCR positive (below the positive threshold of the assay Ct≤27.0) / ELISA (Ingezim and/or Civtest) Seronegative, *(ii)* PCR negative / ELISA Seropositive, and *(iii)* weak PCR+ below the positive threshold (Ct>27.0) / ELISA seropositive. Number of subpatent infections were calculated including all the tests performed (*iv*) and only taking into account weak positives to PCR *(v)* (PCR+ / ELISA + OR -). Non-infected (*vi*) are dogs negative for all the diagnostic tests (PCR-/ ELISA -). (*p<0.05, Fisher’s exact test).

|  |  |  | **Vaccinated** |  |  | **Controls** |  | **Fisher’s exact p, one-**  **tailed** |
| --- | --- | --- | --- | --- | --- | --- | --- | --- |
| **Tp.v.** | **No active infection** | **Nr.** | **Total N/sampling** | **%** | **Nr.** | **Total N/sampling** | **%** |  |
| **T28** | *(i)* PCR+ (Ct>27,0) / ELISA- | 2 | 178 | 1.12 | 3 | 179 | 1.68 | 0.66 |
|  | *(ii)* PCR - / ELISA + | 4 | 178 | 2.25 | 4 | 179 | 2.23 | 0.99 |
|  | *(iii)* PCR+ (Ct>27,0) / ELISA+ | 0 | 178 | 0.00 | 0 | 179 | 0.00 | -- |
|  | *(iv)* Total subpatent infections | 6 | 178 | 3.37 | 7 | 179 | 3.91 | 0.79 |
|  | *(v)* Total subpatent infections (only PCR+) | 2 | 178 | 1.12 | 3 | 179 | 1.68 | 0.50 |
|  | *(vi)* Uninfected (PCR-/ELISA-) | 176 | 178 | 98.88 | 176 | 178 | 98.32 | 0.97 |
| **T194** | PCR+ (Ct>27,0) / ELISA- | 4 | 171 | 2.34 | 3 | 175 | 1.71 | 0.49 |
|  | PCR - / ELISA + | 5 | 171 | 2.92 | 2 | 175 | 1.14 | 0.24 |
|  | PCR+ (Ct>27,0) / ELISA+ | 2 | 171 | 1.17 | 0 | 175 | 0.00 | 0.24 |
|  | Total subpatent infections | 11 | 171 | 6.43 | 5 | 175 | 2.86 | 0.10 |
|  | Total subpatent infections (only PCR+) | 6 | 171 | 3.51 | 3 | 175 | 1.71 | 0.24 |
|  | Uninfected (PCR-/ELISA-) | 164 | 171 | 95.91 | 172 | 175 | 98.29 | 0.87 |
| **T374** | PCR+ (Ct>27.0) / ELISA- | 10 | 162 | 6,17 | 14 | 162 | 8.64 | 0.28 |
|  | PCR - / ELISA + | 3 | 162 | 1.85 | 0 | 162 | 0.00 | 0.12 |
|  | PCR+ (Ct>27,0) / ELISA+ | 1 | 162 | 0.62 | 0 | 162 | 0.00 | 0.50 |
|  | Total subpatent infections | 14 | 162 | 8.64 | 14 | 162 | 8.64 | 0.57 |
|  | Total subpatent infections (only PCR+) | 11 | 162 | 6.79 | 14 | 162 | 8.64 | 0.35 |
|  | Uninfected (PCR-/ELISA-) | 140 | 162 | 86.42 | 139 | 162 | 85.80 | 0.96 |
| **T554** | PCR+ (Ct>27.0) / ELISA- | 4 | 153 | 2.61 | 10 | 151 | 6.62 | 0.092 |
|  | PCR - / ELISA + | 11 | 153 | 7.19 | 4 | 151 | 2.65 | 0.068 |
|  | PCR+ (Ct>27,0) / ELISA+ | 3 | 153 | 1.96 | 3 | 151 | 1.99 | 0.65 |
|  | Total subpatent infections | 18 | 153 | 11.76 | 17 | 151 | 11.26 | 0.52 |
|  | Total subpatent infections (only PCR+) | 7 | 153 | 4.58 | 13 | 151 | 8.61 | 0.13 |
|  | Uninfected (PCR-/ELISA-) | 125 | 153 | 81.70 | 122 | 153 | 79.74 | 0.94 |
| **T644** | PCR+ (Ct>27.0) / ELISA- | 17 | 145 | 11.72 | 11 | 148 | 7.43 | 0.17 |
|  | PCR - / ELISA + | 15 | 145 | 10.34 | 8 | 148 | 5.41 | 0.11 |
|  | PCR+ (Ct>27,0) / ELISA+ | 6 | 145 | 4.14 | 2 | 148 | 1.35 | 0.14 |
|  | Total subpatent infections | 38 | 145 | 26.21 | 21 | 148 | 14.19 | 0.025* |
|  | Total subpatent infections (only PCR+) | 23 | 145 | 15.86 | 13 | 148 | 8.78 | 0.07 |
|  | Uninfected (PCR-/ELISA-) | 105 | 145 | 72.41 | 106 | 148 | 71.62 | 0.95 |
| **T734** | PCR+ (Ct>27.0) / ELISA- | 14 | 141 | 9.93 | 5 | 141 | 3.55 | 0.037* |
|  | PCR - / ELISA + | 12 | 141 | 8.51 | 8 | 141 | 5.67 | 0.38 |
|  | PCR+ (Ct>27,0) / ELISA+ | 10 | 141 | 7.09 | 1 | 141 | 0.71 | 0.0069* |
|  | Total subpatent infections | 36 | 141 | 25.53 | 14 | 141 | 9.93 | 0.029* |
|  | Total subpatent infections (only PCR+) | 24 | 141 | 17.02 | 6 | 141 | 4.26 | 0.0013* |
|  | Uninfected (PCR-/ELISA-) | 103 | 141 | 73.05 | 107 | 141 | 75.89 | 0.45 |
| **LOCF** | PCR+ (Ct>27.0) / ELISA- | 15 | 181 | 8.29 | 7 | 180 | 3.89 | 0.07 |
|  | PCR - / ELISA + | 16 | 181 | 8.84 | 8 | 180 | 4.44 | 0.08 |
|  | PCR+ (Ct>27,0) / ELISA+ | 21 | 181 | 11.60 | 1 | 180 | 0.56 | 0.00* |
|  | Total subpatent infections | 52 | 181 | 28.73 | 16 | 180 | 8.89 | 0.00* |
|  | Total subpatent infections (only PCR+) | 36 | 181 | 19.89 | 8 | 180 | 4.44 | 0.00* |
|  | Uninfected (PCR-/ELISA-) | 127 | 181 | 70.17 | 140 | 180 | 77.78 | 0.52 |
