# Supplementary material for: Large-scale randomized double-blind field clinical trial for safety and efficacy assessment of the DNA vaccine Neoleish against canine leishmaniasis
Source: PLoS Negl Trop Dis. 2025 Nov 3;19(11):e0012707. doi: 10.1371/journal.pntd.0012707 (PMC12604769; doi:10.1371/journal.pntd.0012707)
Supplement: S6 Table — Days 28 and 208 p.v. IFAT titer and diagnostic interpretation, serial indirect ELISAs results, and Ct values of qPCR are showed. For qPCR the positive threshold is Ct ≤ 27.0. (DOCX) [file pntd.0012707.s006.docx]

**S6 Table. Assessment of effect of vaccination on IFAT assay.** Days 28 and 208 p.v. IFAT titer and diagnostic interpretation, serial indirect ELISAs results, and Ct values of qPCR are showed. For qPCR the positive threshold is Ct≤27.0.

| **Dog Id** | **Group** | **T28 p.v.** | | | | | **T208 p.v.** | | | | |
| --- | --- | --- | --- | --- | --- | --- | --- | --- | --- | --- | --- |
|  |  | **IFAT**  **Titer** | **IFAT**  **Result** | **qPCR result (mean Ct value)** | **Ingenasa ELISA Result** | **CivTest ELISA Result** | **IFAT**  **Titer** | **IFAT**  **Result** | **qPCR result (mean Ct value)** | **Ingenasa ELISA Result** | **CivTest ELISA Result** |
| 8712392973 | GA | <1/20 | - | - | - | - | <1/20 | - | - | - | - |
| 8712428341 | GA | <1/20 | - | - | - | - | <1/20 | - | - | - | - |
| 8712393079 | GA | <1/20 | - | - | - | - | <1/20 | - | - | - | - |
| 8712392920 | GA | <1/20 | - | - | - | - | <1/20 | - | - | - | - |
| 8712393001 | GA | <1/20 | - | - | - | - | <1/20 | - | - | - | - |
| 8712392896 | GA | <1/20 | - | - | - | - | <1/20 | - | - | - | - |
| 8712361567 | GA | n.d. | n.d. | n.d. | n.d. | n.d. | <1/20 | - | - | - | - |
| 8712270197 | GA | <1/20 | - | - | - | - | 1/40 | +/- | - | - | - |
| 8711137439 | GA | <1/20 | - | - | - | - | 1/40 | +/- | - | - | - |
| 8711074408 | GA | <1/20 | - | - | - | - | <1/20 | - | - | - | - |
| 938000000337973 | GA | <1/20 | - | - | - | - | <1/20 | - | - | - | - |
| 981098106005425 | GA | <1/20 | - | - | - | - | <1/20 | - | - | - | - |
| 938000000522331 | GA | <1/20 | - | - | - | - | <1/20 | - | - | - | - |
| 941000015294084 | GA | <1/20 | - | - | - | - | <1/20 | - | - | - | - |
| 250268711100229 | GA | <1/20 | - | - | - | - | <1/20 | - | - | - | - |
| 8712392912 | GB | <1/20 | - | - | - | - | <1/20 | - | - | - | - |
| 8712392913 | GB | <1/20 | - | - | - | - | <1/20 | - | - | - | - |
| 8712392953 | GB | <1/20 | - | - | - | - | <1/20 | - | - | - | - |
| 8712392915 | GB | <1/20 | - | - | - | - | <1/20 | - | - | - | - |
| 8712392908 | GB | <1/20 | - | - | - | - | <1/20 | - | 37.55 | - | - |
| 8712360909 | GB | <1/20 | - | - | - | - | <1/20 | - | - | - | - |
| 8712292946 | GB | <1/20 | - | - | - | - | <1/20 | - | - | - | - |
| 938000000371011 | GB | <1/20 | - | - | - | - | <1/20 | - | - | - | - |
| 981098106002804 | GB | <1/20 | - | - | - | - | n.d. | n.d. | n.d. | n.d. | n.d. |
| 250268720036515 | GB | <1/20 | - | - | - | - | <1/20 | - | - | - | - |
| 981098106010638 | GB | <1/20 | - | - | - | - | <1/20 | - | - | - | - |
| 941000016440434 | GB | <1/20 | - | - | - | - | <1/20 | - | - | - | - |
| 938000000305964 | GB | <1/20 | - | - | - | - | <1/20 | - | - | - | - |
| 250268711100207 | GB | <1/20 | - | - | - | - | <1/20 | - | - | - | - |
| 941000016440441 | GB | <1/20 | - | - | - | - | <1/20 | - | - | - | - |

**S6 Table** (continued)

| **Dog Id** | **Group** | **T388 p.v.** | | | | | **T573 p.v.** | | | | |
| --- | --- | --- | --- | --- | --- | --- | --- | --- | --- | --- | --- |
|  |  | **IFAT**  **Titer** | **IFAT**  **Result** | **PCR result (mean Ct value)** | **Ingenasa ELISA Result** | **CivTest ELISA Result** | **IFAT**  **Titer** | **IFAT**  **Result** | **PCR result (mean Ct value)** | **Ingenasa ELISA Result** | **CivTest ELISA Result** |
| 8712392973 | GA | <1/20 | - | - | - | - | <1/20 | - | - | - | - |
| 8712428341 | GA | <1/20 | - | - | - | - | <1/20 | - | - | - | +L |
| 8712393079 | GA | <1/20 | - | 36.37 | - | - | <1/20 | - | 37.36 | - | - |
| 8712392920 | GA | 1/40 | +/- | 27.73 | - | - | 1/640 | + | 16.37 | + | +H |
| 8712393001 | GA | <1/20 | - | 22.82 | - | - | 1/1280 | + | 18.07 | + | +H |
| 8712392896 | GA | <1/20 | - | - | - | - | <1/20 | - | - | - | - |
| 8712361567 | GA | <1/20 | - | - | - | - | 1/40 | +/- | - | - | - |
| 8712270197 | GA | <1/20 | - | - | - | - | 1/40 | +/- | - | - | - |
| 8711137439 | GA | <1/20 | - | - | - | - | 1/40 | +/- | - | - | - |
| 8711074408 | GA | 1/40 | +/- | 26.83 | - | - | 1/320 | + | 16.87 | + | +H |
| 938000000337973 | GA | <1/20 | - | - | - | - | <1/20 | - | - | - | - |
| 981098106005425 | GA | <1/20 | - | - | - | - | 1/40 | +/- | - | - | - |
| 938000000522331 | GA | <1/20 | - | - | - | - | <1/20 | - | - | - | - |
| 941000015294084 | GA | 1/40 | +/- | - | - | - | 1/40 | +/- | 33.50 | - | - |
| 250268711100229 | GA | <1/20 | - | - | - | - | <1/20 | - | - | - | - |
| 8712392912 | GB | <1/20 | - | 34.36 | - | - | <1/20 | - | - | - | - |
| 8712392913 | GB | <1/20 | - | - | - | - | <1/20 | - | - | - | - |
| 8712392953 | GB | <1/20 | - | 32.30 | - | - | 1/40 | +/- | - | - | - |
| 8712392915 | GB | <1/20 | - | - | - | - | <1/20 | - | - | - | - |
| 8712392908 | GB | 1/1280 | + | 13.42 | - | - | 1/2560 | + | 21.65 | + | +H |
| 8712360909 | GB | 1/20 | - | - | - | - | <1/20 | - | - | - | - |
| 8712292946 | GB | 1/40 | +/- | 30.88 | - | - | 1/40 | +/- | - | - | - |
| 938000000371011 | GB | <1/20 | - | 31.49 | - | - | 1/40 | +/- | 28.43 | - | - |
| 981098106002804 | GB | n.d. | n.d. | n.d. | n.d. | n.d. | n.d. | n.d. | n.d. | n.d. | n.d. |
| 250268720036515 | GB | <1/20 | - | - | - | - | <1/20 | - | - | - | - |
| 981098106010638 | GB | <1/20 | - | - | - | - | 1/40 | +/- | 28.95 | - | - |
| 941000016440434 | GB | <1/20 | - | - | - | - | 1/40 | +/- | - | - | - |
| 938000000305964 | GB | <1/20 | - | - | - | - | n.d. | n.d. | n.d. | n.d. | n.d. |
| 250268711100207 | GB | <1/20 | - | - | - | - | <1/20 | - | - | - | - |
| 941000016440441 | GB | 1/40 | +/- | - | - | - | <1/20 | - | - | - | - |

**S6 Table** (continued)

| **Dog Id** | **Group** | **T644 p.v.** | | | | | **T748 p.v.** | | | | |
| --- | --- | --- | --- | --- | --- | --- | --- | --- | --- | --- | --- |
|  |  | **IFAT**  **Titer** | **IFAT**  **Result** | **PCR result (mean Ct value)** | **Ingenasa ELISA Result** | **CivTest ELISA Result** | **IFAT**  **Titer** | **IFAT**  **Result** | **PCR result (mean Ct value)** | **Ingenasa ELISA Result** | **CivTest ELISA Result** |
| 8712392973 | GA | <1/20 | - | 34.52 | - | - | <1/20 | - | - | - | - |
| 8712428341 | GA | <1/20 | - | - | - | - | <1/20 | - | - | - | - |
| 8712393079 | GA | <1/20 | - | - | - | - | <1/20 | - | - | - | - |
| 8712392920 | GA | 1/1280 | + | 17.79 | + | +H | 1/1280 | + | 30.16 | + | +H |
| 8712393001 | GA | 1/2560 | + | 15.70 | + | +H | 1/640 | + | 26.17 | + | +H |
| 8712392896 | GA | 1/40 | +/- | - | - | - | <1/20 | - | - | - | - |
| 8712361567 | GA | <1/20 | - | 34.60 | - | - | <1/20 | - | - | - | - |
| 8712270197 | GA | 1/20 | - | - | - | - | <1/20 | - | - | - | - |
| 8711137439 | GA | 1/40 | +/- | - | - | - | <1/20 | - | - | - | - |
| 8711074408 | GA | 1/640 | + | 16.05 | + | +H | 1/640 | + | 20.03 | + | +H |
| 938000000337973 | GA | <1/20 | - | 26.33 | - | - | <1/20 | - | - | - | - |
| 981098106005425 | GA | n.d. | n.d. | n.d. | n.d. | n.d. | n.d. | n.d. | n.d. | n.d. | n.d. |
| 938000000522331 | GA | <1/20 | - | 36.28 | - | - | <1/20 | - | 39.05 | - | - |
| 941000015294084 | GA | <1/20 | - | 35.57 | - | - | <1/20 | - | 38.33 | - | - |
| 250268711100229 | GA | <1/20 | - | 38.98 | - | - | <1/20 | - | - | - | - |
| 8712392912 | GB | <1/20 | - | - | - | - | <1/20 | - | - | - | - |
| 8712392913 | GB | <1/20 | - | 39.22 | - | - | <1/20 | - | - | - | - |
| 8712392953 | GB | 1/320 | + | 15.15 | + | + | 1/1280 | + | 17.73 | +/- | +H |
| 8712392915 | GB | <1/20 | - | - | - | - | <1/20 | - | - | - | - |
| 8712392908 | GB | 1/2560 | + | 14.84 | + | +H | 1/2560 | + | 19.01 | + | +H |
| 8712360909 | GB | <1/20 | - | - | - | - | <1/20 | - | - | - | - |
| 8712292946 | GB | <1/20 | - | 26.48 | - | - | 1/40 | +/- | 21.78 | - | - |
| 938000000371011 | GB | <1/20 | - | 23.01 | - | +/- | 1/40 | +/- | 20.39 | + | + |
| 981098106002804 | GB | n.d. | n.d. | n.d. | n.d. | n.d. | n.d. | n.d. | n.d. | n.d. | n.d. |
| 250268720036515 | GB | <1/20 | - | 36.22 | - | - | <1/20 | - | 37.13 | - | - |
| 981098106010638 | GB | n.d. | n.d. | n.d. | n.d. | n.d. | n.d. | n.d. | n.d. | n.d. | n.d. |
| 941000016440434 | GB | 1/160 | + | 12.11 | + | +H | 1/1280 | + | 22.53 | + | +H |
| 938000000305964 | GB | n.d. | n.d. | n.d. | n.d. | n.d. | n.d. | n.d. | n.d. | n.d. | n.d. |
| 250268711100207 | GB | <1/20 | - | 36.88 | - | - | <1/20 | - | - | - | - |
| 941000016440441 | GB | <1/20 | - | 35.97 | - | - | <1/20 | - | - | - | - |
