# Supplementary material for: Large-scale randomized double-blind field clinical trial for safety and efficacy assessment of the DNA vaccine Neoleish against canine leishmaniasis
Source: PLoS Negl Trop Dis. 2025 Nov 3;19(11):e0012707. doi: 10.1371/journal.pntd.0012707 (PMC12604769; doi:10.1371/journal.pntd.0012707)
Supplement: S7 Table — *Pregnancy periods, 1st: from 0 to 22 days of gestation, 2nd: 23–44, 3rd: 45–65). (DOCX) [file pntd.0012707.s007.docx]

**S7 Table. Pregnant females treated during the study**. *Pregnancy periods, 1^st^: from 0 to 22 days of gestation, 2^nd^: 23-44, 3^rd^: 45-65.

| **Kennel id.** | **Dog Id.** | **Repeated dose** | **Treat.** | **Date of parturition** | **Days of gestation during treatment** | **Stage of gestatio n*** | **Litter size (Nr.)** |
| --- | --- | --- | --- | --- | --- | --- | --- |
| 2-CC | 941000021077223 | No | GA | 20-Nov-18 | 3 | 1 | 8 |
| 6-BA | 941000018923019 | No | GA | 08-Apr-19 | 58 | 3 | 8 |
| 6-BA | 981100002547693 | No | GA | 05-Apr-17 | 52 | 3 | 9 |
| 1-BA | 941000018975220 | No | GA | 14-May-18 | 10 | 1 | 8 |
| 1-BA | 941000018975220 | Yes | GA | 28-May-19 | 10 | 1 | 9 |
| 1-BA | 941000018975303 | No | GA | 01-Jun-17 | 8 | 1 | 10 |
| 1-BA | 941000013631514 | No | GA | 05-Oct-17 | 49 | 3 | 7 |
| 1-BA | 941000018464440 | No | GA | 02-Apr-17 | 54 | 3 | 5 |
| 1-BA | 941000018464440 | Yes | GA | 15-May-18 | 11 | 1 | 8 |
| 7-BA | 941000012777125 | No | GB | 20-Apr-17 | 50 | 3 | 8 |
| 3-CC | 941000019511095 | No | GB | 04-Oct-17 | 58 | 3 | 7 |
| 2-CC | 939000010095383 | No | GB | 27-Apr-17 | 50 | 3 | 5 |
| 2-CC | 941000021077181 | No | GB | 20-Mar-18 | 58 | 3 | 8 |
| 6-BA | 981098104808051 | No | GB | 26-Mar-17 | 62 | 3 | 7 |
| 6-BA | 941000018418517 | No | GB | 07-Apr-17 | 63 | 3 | 6 |
| 1-BA | 941000018249082 | No | GB | 18-Apr-19 | 50 | 3 | 8 |
| 1-BA | 941000018249587 | No | GB | 18-Apr-19 | 50 | 3 | 8 |
| 1-BA | 941000016101056 | No | GB | 18-Apr-19 | 50 | 3 | 9 |
| 1-BA | 941000012306785 | No | GB | 29-Mar-17 | 58 | 3 | 10 |
| 1-BA | 941000012306785 | Yes | GB | 15-May-18 | 9 | 1 | 7 |
| 4-CC | 941000014586993 | No | GB | 04-Oct-17 | 58 | 3 | 8 |
