# Supplementary material for: Large-scale randomized double-blind field clinical trial for safety and efficacy assessment of the DNA vaccine Neoleish against canine leishmaniasis
Source: PLoS Negl Trop Dis. 2025 Nov 3;19(11):e0012707. doi: 10.1371/journal.pntd.0012707 (PMC12604769; doi:10.1371/journal.pntd.0012707)
Supplement: S8 Table — (DOCX) [file pntd.0012707.s008.docx]

**S8 Table. Summary of litter sizes by treatment group and number of pregnant females treated during the study, broken down by term of pregnancy.**

|  | **First third of gestation** | | **Last third of gestation** | | **All gestations** | |
| --- | --- | --- | --- | --- | --- | --- |
| **Treatment group** | **Mean litter size** | **Nr.** | **Mean litter size** | **Nr.** | **Mean litter size** | **Nr.** |
| Vaccinated* (GA) | 8.6 | 5.0 | 7.3 | 4.0 | 8.0 | 9.0 |
| Controls (GB) | 7.0 | 1.0 | 7.6 | 11.0 | 7.6 | 12.0 |
| **Total** | **8.3** | **6.0** | **7.5** | **15.0** | **7.8** | **21.0*** |

*Repeated vaccinations have been included as independent events
